# Multi‐omics analysis of disulfidptosis regulators and therapeutic potential reveals glycogen synthase 1 as a disulfidptosis triggering target for triple‐negative breast cancer

**DOI:** 10.1002/mco2.502

**Published:** 2024-02-28

**Authors:** Jindong Xie, Xinpei Deng, Yi Xie, Hongbo Zhu, Peng Liu, Wei Deng, Li Ning, Yuhui Tang, Yuying Sun, Hailin Tang, Manbo Cai, Xiaoming Xie, Yutian Zou

**Affiliations:** ^1^ State Key Laboratory of Oncology in South China Guangdong Provincial Clinical Research Center for Cancer Sun Yat‐Sen University Cancer Center Guangzhou Guangdong China; ^2^ The First Affiliated Hospital Hengyang Medical School University of South China Hengyang Hunan China

**Keywords:** disulfidptosis, pan‐cancer, prognosis, single‐cell RNA‐seq, tumor microenvironment

## Abstract

Disruption of disulfide homeostasis during biological processes can have fatal consequences. Excess disulfides induce cell death in a novel manner, termed as “disulfidptosis.” However, the specific mechanism of disulfidptosis has not yet been elucidated. To determine the cancer types sensitive to disulfidptosis and outline the corresponding treatment strategies, we firstly investigated the crucial functions of disulfidptosis regulators pan‐cancer at multi‐omics levels. We found that different tumor types expressed dysregulated levels of disulfidptosis regulators, most of which had an impact on tumor prognosis. Moreover, we calculated the disulfidptosis activity score in tumors and validated it using multiple independent datasets. Additionally, we found that disulfidptosis activity was correlated with classic biological processes and pathways in various cancers. Disulfidptosis activity was also associated with tumor immune characteristics and could predict immunotherapy outcomes. Notably, the disulfidptosis regulator, glycogen synthase 1 (*GYS1*), was identified as a promising target for triple‐negative breast cancer and validated via in vitro and in vivo experiments. In conclusion, our study elucidated the complex molecular phenotypes and clinicopathological correlations of disulfidptosis regulators in tumors, laying a solid foundation for the development of disulfidptosis‐targeting strategies for cancer treatment.

## Graphical abstract

Flowchart of our study. By utilizing multi‐omics pan‐cancer cohorts, our study firstly offers a pan‐cancer blueprint of the molecular and clinical characteristics of disulfidptosis regulators, as wells as disulfidptosis activity, which lay a solid foundation for the disulfidptosis‐targeting strategy in precision cancer treatment.

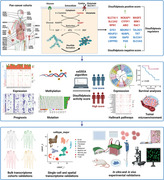

## 1 INTRODUCTION

Imbalance in disulfide homeostasis may be fatal. A distinct type of cell death caused by disulfide accumulation has recently been characterized as “disulfidptosis.”^1^ Unlike apoptosis, ferroptosis, or any other type of previously reported cell death,^2^, ^3^ disulfidptosis is mediated by the vulnerability of actin cytoskeleton to disulfide‐induced stress. During disulfidation, misfolded and aggregated proteins are selectively destroyed by the proteasomal system via oxidation‐dependent process. Disulfide metabolism is closely correlated with tumors. Disulfide metabolism refers to intracellular redox reactions involving the formation and breakdown of disulfide bonds. According to previous studies, tumor cells may undergo metabolic reprogramming, leading to an increase in oxidative stress, which causes disulfide metabolism disorder and impacts their survival and proliferation.^4^, ^5^, ^6^, ^7^ Additionally, disulfide metabolism influences the metastasis, drug resistance, and immune escape of tumor cells.^8^, ^9^, ^10^ Liu et al. further conducted a CRISPR/Cas9 loss‐of‐function screening and reported candidate agents participating in disulfidptosis.^11^, ^12^ We defined them as disulfidptosis regulators based on their roles in regulating disulfidptosis activity. Among the reported candidates, nine genes were positive (*SLC7A11*, *SLC3A2*, *RPN1*, *NCKAP1*, *CYFIP1*, *ABI2*, *WASF2*, *BRK1*, and *RAC1*), and the others were negative (*GYS1*, *NDUFS1*, *OXSM*, *LRPPRC*, *NDUFA11*, *NUBPL*, *G6PD*, *PGD*, *TALDO1*, *TKT*, *SLC2A1*, and *SLC2A3*) regulators of disulfidptosis activity. Further exploration of these regulators will help in understanding the mechanisms regulating this newly identified form of cell death.

Previous studies have explored molecular and clinical characteristics of various forms of cell death in tumors, and disulfidptosis‐based studies have been studied in oncological and non‐oncological diseases at multi‐omics levels.^13^, ^14^, ^15^, ^16^ Novel therapeutic targets can be identified and personalized treatment strategies can be developed mechanism of disulfidptosis. It is imperative for future research to focus on potential action mechanisms of disulfidptosis regulators in tumor development to improve patient outcomes.

This study provides a preliminary overview and reference point for future research on disulfidptosis. We utilized multi‐omics pan‐cancer cohorts to comprehensively evaluate the molecular and clinical characteristics of disulfidptosis regulators. Notably, the disulfidptosis regulator, *GYS1*, was identified as a promising target for triple‐negative breast cancer (TNBC) and validated via in vitro and in vivo experiments.

*GYS1*, situated at the chromosomal locus 19q13.3, serves as the primary modulator of glycogen synthesis in extrahepatic tissues.^17^, ^18^ This gene's protein adds glucose monomers to glycogen by forming α‐1,4‐glycoside linkages. It connects the carbon‐1 of UDP‐glucose to the carbon‐4 of glycogen.^19^ *GYS1* serves as the key rate‐limiting enzyme in glycogen synthesis,^20^ and its deficiency causes muscle glycogen storage disease type 0 and death at a pathological level.^21^, ^22^ Besides, previous studies have found that targeting the *GYS1* could halt the progression of epilepsy and neuroinflammation.^23^, ^24^, ^25^ Additionally, numerous studies have shown that *GYS1* participates in tumor growth and progression through different mechanisms such as via the nuclear factor kappa‐B (NF‐κB) pathway, the AMP‐activated protein kinase (AMPK) pathway, and the hypoxic inducible factor‐1 alpha (HIF‐1α) pathway.^26^, ^27^, ^28^ The enzymatic function of *GYS1* can be regulated through post‐translational modification and the influence of allosteric effectors. Specifically, phosphorylation induced by glucagon and epinephrine hormones results in the inactivation of *GYS1*, while dephosphorylation triggered by insulin hormone exerts an activating effect on *GYS1*.^29^ Our results revealed the crucial roles of disulfidptosis in tumor growth and progression, indicating the potential of disulfidptosis‐targeting strategies for cancer treatment.

## 2 RESULTS

### 2.1 Disulfidptosis regulators exhibited different gene expression patterns in various tumor types

**FIGURE 1:**
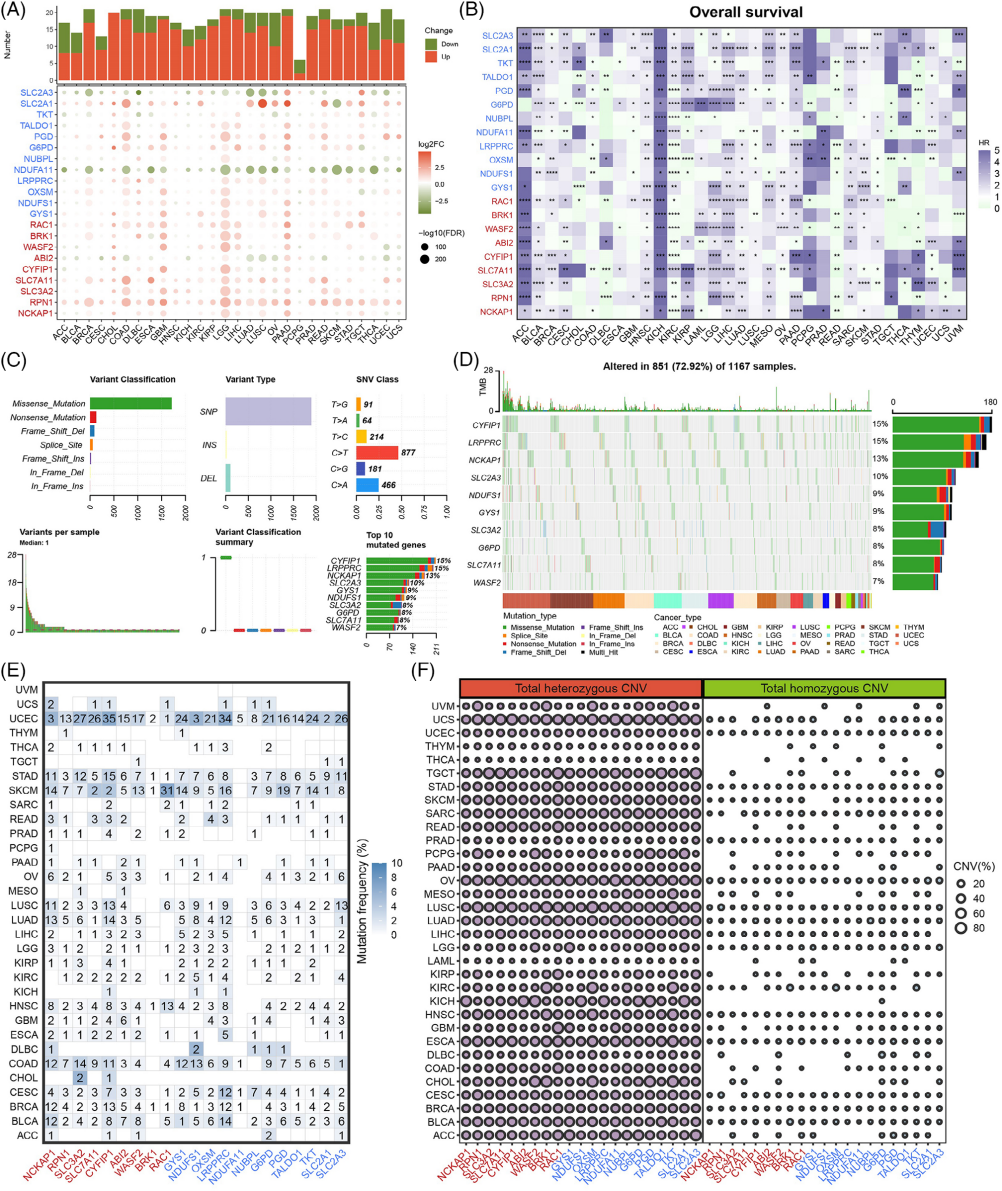
Gene expression, prognosis, and mutation patterns of disulfidptosis regulators. (A) Gene expression patterns of disulfidptosis regulators in pan‐cancer cohorts. Histogram (upper panel) shows the number of significantly differentially expressed disulfidptosis regulators. Upregulated and downregulated genes are marked in red and green, respectively (false discovery rate [FDR]; fold change [FC]). (B) Prognosis patterns of disulfidptosis regulators in The Cancer Genome Atlas (TCGA) pan‐cancer cohorts. Risky and protective genes are marked in purple and green, respectively (hazard ratio [HR]; ^*^ *p* < 0.05, ^**^ *p* < 0.01, ^***^ *p* < 0.001, ^****^ *p* < 0.0001). (C) The single nucleotide variation (SNV) classes of disulfidptosis regulators in pan‐cancer cohorts. (D) Oncoplot of the mutation distribution of disulfidptosis regulators in pan‐cancer. (E) The SNV profile of disulfidptosis regulators in each tumor type. (F) The heterozygous and homozygous copy number variation (CNV) profile of disulfidptosis regulators in each tumor type.

We examined the relationships among the disulfidptosis regulators. We observed that 21 regulators generated interaction links (Figure S1). We explored the expression levels of each disulfidptosis regulator in normal and tumor tissues of pan‐cancer cohorts from The Cancer Genome Atlas (TCGA) and the Genotype‐Tissue Expression (GTEx) dataset. Figure 1A shows that the expression levels of disulfidptosis regulators in most tumors were aberrant. *GYS1*, *RAC1*, *SLC7A11*, and *RPN1* levels were mostly upregulated, whereas *NDUFA11*, *ABI2*, and *SLC2A3* levels were mostly downregulated. The other regulators showed heterogeneous expression patterns. For instance, *SLC2A1* levels were upregulated in most tumors but significantly downregulated in skin cutaneous melanoma. Besides, we explored the expression and distribution of each disulfidptosis regulator using TISCH database, and we found that *BRK1*, *G6PD*, *NDUFA11*, and *RAC1* are highly expressed in most cell types (Figure S2). These findings suggest that disulfidptosis activity is correlated with the expression patterns of disulfidptosis regulators in tumors.

### 2.2 Disulfidptosis regulators were closely related to the prognosis of certain tumor types

Next, we evaluated the prognostic patterns of disulfidptosis regulators. We selected four prognostic indicators, namely overall survival (OS), disease‐free survival (DFS), progression‐free survival (PFS), and disease‐specific survival (DSS), from TCGA datasets. The results showed that disulfidptosis regulators were related to patient prognoses in multiple tumors (Figures 1B and S3). Their prognostic roles varied widely in different cancer types. For example, *SLC7A11* was a risky factor for OS in breast cancer (BRCA) but a protective factor in colon adenocarcinoma (COAD). Moreover, the roles of disulfidptosis regulators as prognostic indicators were observed to be heterogeneous. For instance, in bladder cancer (BLCA), *OXSM* was a risk factor for DFS and a protective factor for OS, DSS, and PFS. Interestingly, disulfidptosis regulators acted as risk factors for adrenocortical cancer, kidney chromophobe, and sarcoma but as prognostic factors in rectum adenocarcinoma. These results indicate that the disulfidptosis regulators are closely related to the prognosis of certain tumor types.

### 2.3 Mutation and methylation landscape of disulfidptosis regulators

We investigated the genomic alterations in disulfidptosis regulators using copy number variation (CNV) and single nucleotide variation (SNV) data from TCGA cohorts. Generally, *CYFIP1* and *LRPPRC* showed the highest SNV frequencies among the disulfidptosis regulators (15%), and most disulfidptosis regulators had high SNV frequencies in tumors (Figure 1C,D). Additionally, most mutations were missense mutations. Furthermore, we examined the somatic alterations in each cancer type to obtain better understandings of mutation landscape. Disparate mutation patterns were observed in different tumor types (Figure 1E). All disulfidptosis regulators exhibited mutations in cervical cancer (CESC), whereas no mutations were observed in ocular melanomas (UVM). Additionally, disulfidptosis regulators exhibited the highest average SNV frequency in patients with uterine corpus endometrial carcinoma (UCEC). For CNV patterns, we analyzed each disulfidptosis regulator in different tumor types. We found that the CNV profiles of disulfidptosis regulators were higher in the heterozygous region than in the homozygous region (Figures 1F and S4). Moreover, we analyzed the correlations between the CNV profiles and disulfidptosis regulator expression levels (Figure S5).

**FIGURE 2:**
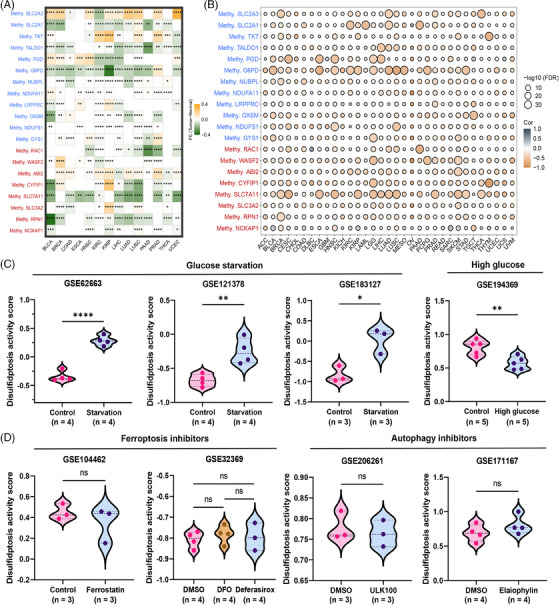
Methylation level of disulfidptosis regulators, and establishment of the disulfidptosis activity score. (A) Heatmap of the different methylation levels of disulfidptosis regulators in pan‐cancer. Upregulated and downregulated genes are marked in yellow and green, respectively (fold change [FC]; ^*^ *p* < 0.05, ^**^ *p* < 0.01, ^***^ *p* < 0.001, ^****^ *p* < 0.0001). (B) Bubble plot of the correlations between the methylation level and mRNA expression of disulfidptosis regulators in pan‐cancer. Positive and negative correlations are marked in gray and yellow, respectively (false discovery rate [FDR]). (C) Violin plot showed the different disulfidptosis activity scores between control condition and glucose starvation (or high glucose) in GSE62663, GSE121378, GSE183127, and GSE194369. ^*^ *p* < 0.05, ^**^ *p* < 0.01, ^****^ *p* < 0.0001. (D) Violin plot showed the different disulfidptosis activity scores between control condition and multiple types of inhibitors in GSE104462, GSE32369, GSE206261, and GSE171167 (ns: no significance).

Methylation plays vital roles in the gene expression regulation, and hypermethylation suppresses gene expression.^30^, ^31^ However, hypermethylation may increase the gene expression in some cases.^32^ Here, we investigated whether the methylation levels of disulfidptosis regulators differed between tumor and paired normal tissues. Notably, we found differences across all 14 tumors, and the methylation patterns were heterogeneous (Figure 2A). For example, *SLC2A3* was hypermethylated in BLCA, BRCA, head and neck squamous cell carcinoma (HNSC), kidney renal papillary cell carcinoma, liver hepatocellular carcinoma, lung adenocarcinoma, lung squamous cell carcinoma (LUSC), prostate adenocarcinoma (PRAD), and UCEC but hypomethylated in COAD and kidney renal clear cell carcinoma (KIRC). Interestingly, a consistent reduction in *SLC7A11* methylation was observed in tumor tissues. Then, we calculated correlations between methylation and the expression levels of disulfidptosis regulators. As shown in Figure 2B, methylation levels were generally negatively related to corresponding expression levels, except for *NCKAP1*, *RAC1*, *PGD*, and *SLC2A1*, which exhibited positive correlations. These results disclose that the methylation levels of disulfidptosis regulators might affect tumor progression.

### 2.4 Establishment and validation of the disulfidptosis activity score

To better understand their potential roles in pan‐cancer cohorts, we conducted single‐sample gene set enrichment analysis (ssGSEA) to calculate the scores of disulfidptosis suppressors and activators. We defined them as “disulfidptosis‐negative score” and “disulfidptosis‐positive score,” and further calculated the disulfidptosis activity score. Nine independent datasets were collected from Gene Expression Omnibus (GEO) database to verify the established disulfidptosis activity score. Compared to tumor cells cultured under normal conditions, tumor cells under glucose starvation exhibited significantly higher disulfidptosis activity scores, whereas those under high glucose conditions exhibited lower disulfidptosis activity scores (Figures 2C and S6). Moreover, Figure 2D shows that addition of ferroptosis or autophagy inhibitors did not affect the disulfidptosis activity score. These results suggest that disulfidptosis activity score reflects the general disulfidptosis activity levels in tumor cells.

### 2.5 Pathway activity analyses and immune characteristics of the disulfidptosis activity score

**FIGURE 3:**
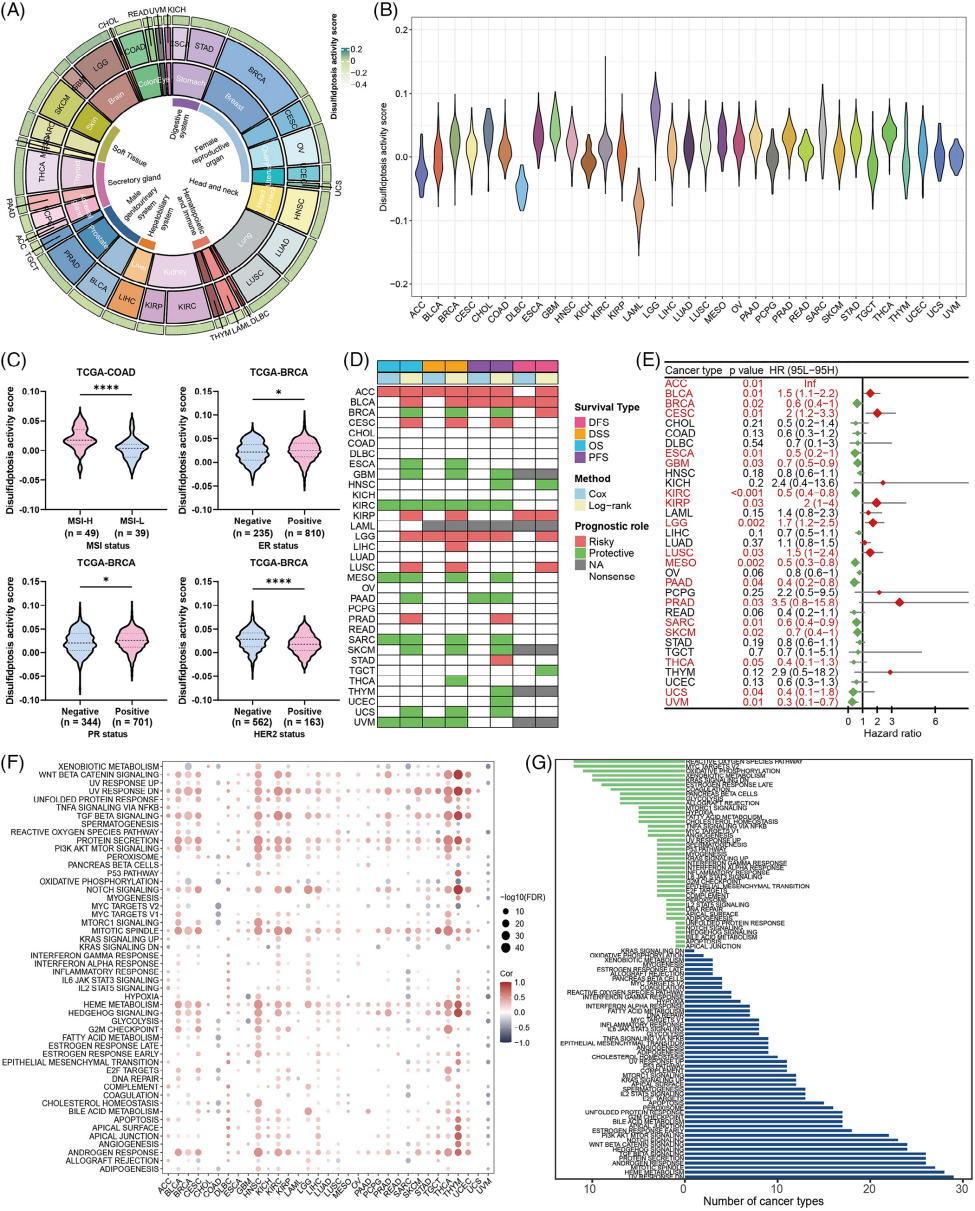
Expression, prognosis, and correlated hallmark pathways of the disulfidptosis activity score. (A) Comprehensive quantification of the disulfidptosis activity score in pan‐cancer. Tissue types, cancer types and average disulfidptosis activity scores are shown from the inner circle to the outer circle. (B) Violin plot showed the disulfidptosis activity score level in pan‐cancer. (C) Violin plots showed the different disulfidptosis activity scores between microsatellite instability (MSI), estrogen receptor (ER), progesterone receptor (PR), and human epidermal growth factor receptor‐2 (HER2) statuses in The Cancer Genome Atlas (TCGA)‐colon adenocarcinoma (COAD) and TCGA‐breast cancer (BRCA) cohorts. (D) Prognostic value of the disulfidptosis activity score in pan‐cancer using univariate Cox and log‐rank regression methods. (E) The forest plot exhibited the prognostic role of the disulfidptosis activity score in pan‐cancer by univariate Cox regression method. The cancer type in red represents the disulfidptosis activity score acts as a prognostic factor with statistical significance. (F) Bubble plot of the correlations between the disulfidptosis activity score and hallmark pathways in pan‐cancer. Positive and negative correlations are marked in red and blue, respectively (false discovery rate [FDR]). (G) Bar plot showing the number of cancer types having negative (green) and positive (blue) correlations between disulfidptosis activity and hallmark pathways.

According to our findings, disulfidptosis is dysregulated and crucial for tumor prognosis. Therefore, we first comprehensively quantified the disulfidptosis activity score in pan‐cancer cells and found that different tumor types exhibited different disulfidptosis activities (Figure 3A). Among the pan‐cancer cohorts, lower grade glioma (LGG) showed the highest average and lymphoblastic acute myeloid leukemia showed the lowest disulfidptosis activity score (Figure 3B). In TCGA‐COAD cohort, the disulfidptosis activity scores in the high microsatellite instability (MSI‐H) group were higher than those in low microsatellite instability (MSI‐L) group. Moreover, the estrogen receptor, progesterone receptor, and human epidermal growth factor receptor‐2 statuses exhibited different disulfidptosis activity scores (Figure 3C). We further explored the prognostic value of the disulfidptosis activity score for each tumor type using univariate COX and log‐rank regression analyses. Notably, the disulfidptosis activity score played a protective role in most tumor types (Figure 3D,E). However, the specific tumor‐related pathways involved in disulfidptosis remain unknown. Therefore, we analyzed 50 classic cancer hallmark gene sets to determine their enrichment levels in each sample. Next, we computed their correlations with the disulfidptosis activity scores. The result showed that disulfidptosis activity was mainly positively associated with hallmark gene sets in pan‐cancer analysis (Figure 3F). Interestingly, a significant negative correlation was observed between the reactive oxygen species pathway and disulfidptosis activity in most tumor types. In addition, the reactive oxygen species pathway and MYC targets v2 were negatively correlated, whereas ultraviolet response signaling, heme metabolism, and mitotic spindles were most positively correlated (Figure 3G). Taken together, these findings suggest that the disulfidptosis activity score affects multiple biological processes and reliably reflects the disulfidptosis level in tumors.

**FIGURE 4:**
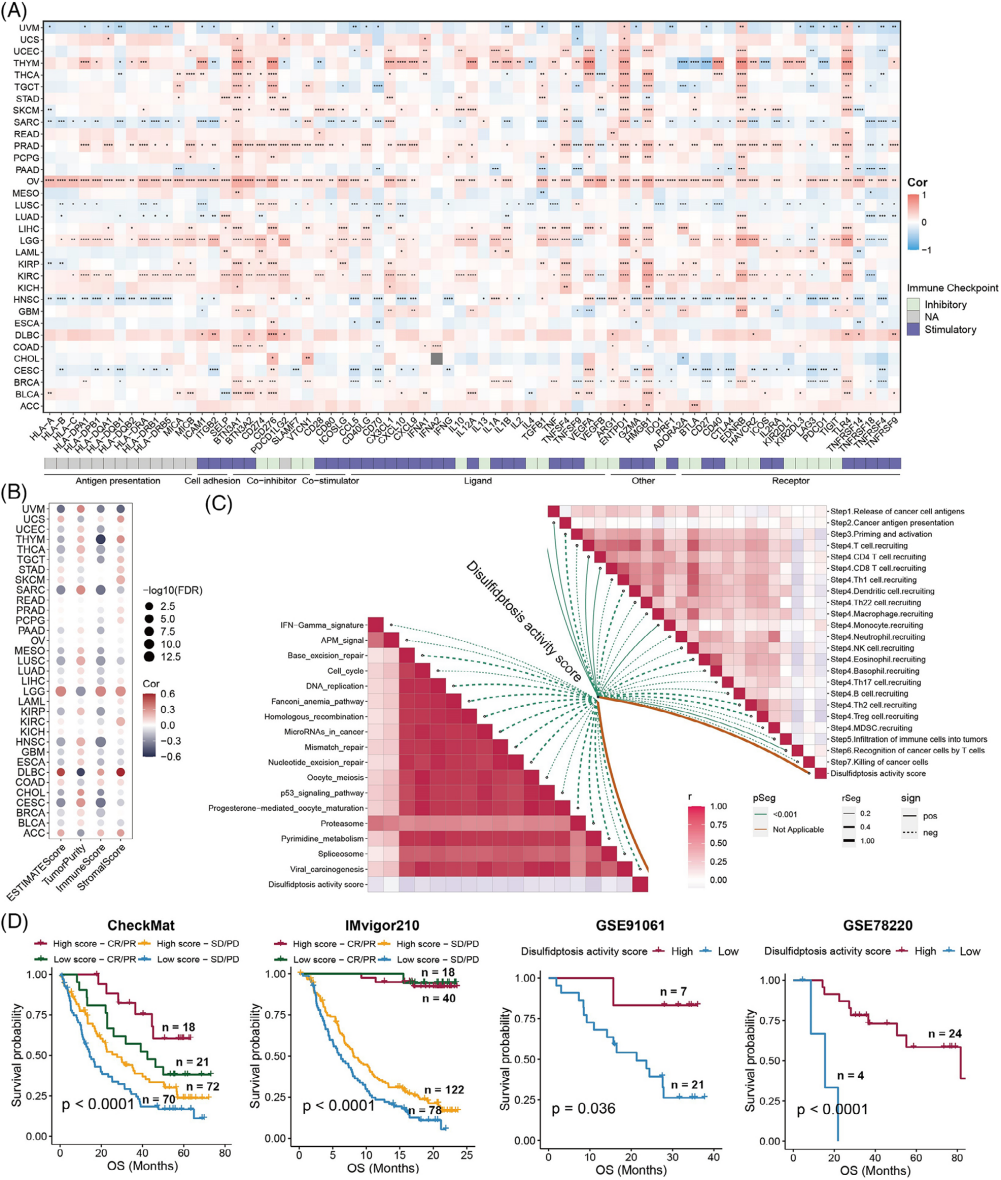
Disulfidptosis activity score correlates with tumor immune characteristics. (A) Heatmap of the correlations between the disulfidptosis activity score and immune checkpoints in each tumor type. Positive and negative correlations are marked in red and blue, respectively. ^*^ *p* < 0.05, ^**^ *p* < 0.01, ^***^ *p* < 0.001, ^****^ *p* < 0.0001. (B) Bubble plot of the correlations between the disulfidptosis activity score and ESTIMATEScore, TumorPurity, ImmuneScore, and StromalScore in pan‐cancer. Positive and negative correlations are marked in red and blue, respectively (false discovery rate [FDR]). (C) The lower left panel shows the correlations between the disulfidptosis activity score and immunoregulation‐related pathways. The upper right panel shows the correlations between the disulfidptosis activity score and cancer immunity cycles. (D) The Kaplan–Meier curve showing the difference in overall survival (OS) time between high‐ and low‐disulfidptosis activity score in multiple immunotherapy cohorts. High‐disulfidptosis activity score is indicated in the red line and low‐disulfidptosis activity score in blue.

We then measured the expression levels of classic immune checkpoints and calculated their correlations with the disulfidptosis activity scores. The disulfidptosis activity score was found correlated with various immune checkpoints (Figure 4A). Notably, relatively more positive correlations in KIRC, LGG, ovarian cancer, and PRAD, and more negative correlations were observed in CESC, HNSC, LUSC, and UVM. Moreover, the ESTIMATE algorithm was used to infer the overall stromal and immune infiltration levels,^33^ and the findings were broadly consistent with the above (Figure 4B). We found that most tumor types had significant negative correlations between disulfidptosis activity and immune and stromal cell infiltration. We also constructed a heatmap demonstrating the infiltration levels of multiple cells using different algorithms (Figure S7).^34^ Figure 4C shows that the disulfidptosis activity score is negatively correlated with most cancer immunity cycles procedures and exhibited different correlations with immune‐related functions.

Immune checkpoint inhibitors are the most commonly used and successful immunotherapeutic agents.^35^, ^36^, ^37^ After the determination of the associations between disulfidptosis activity and numerous immune‐related characteristics, several immunotherapy cohorts were used to explore whether disulfidptosis activity affects the immunotherapy outcomes of patients with tumors. As shown in Figure 4D, a high degree of disulfidptosis activity was related to a better prognosis after immunotherapy. Taken together, disulfidptosis activity score might be predictive of immunotherapy efficacy in patients with tumors.

### 2.6 Determination of disulfidptosis activity score in scRNA‐seq cohorts

Tumor cells can be accurately identified by scRNA‐seq technology both intrinsically and extrinsically. It can identify various cell types, illustrating clonal diversity and critical factors influencing tumor heterogeneity.^38^, ^39^ Here, we determined the disulfidptosis activity score in several scRNA‐seq cohorts. First, we reduced the dimensions of each cohort and colored the different cell types (Figure S8A). The disulfidptosis activity score of each cell was assessed, and violin plots demonstrated that in epithelial cells, the disulfidptosis activity score of malignant cells was consistently lower than that of normal cells, whereas endothelial cells appeared higher disulfidptosis activity scores in each cohort (Figure S8B). Moreover, feature plots confirmed that most cells basically possessed a high‐disulfidptosis activity score (Figure S8C). Subsequently, we separated malignant cells into activated and suppressive clusters based on the median values of the disulfidptosis activity score, and performed cell–cell communication analysis in each cohort. Similarly, malignant cells in activated clusters generally communicated more with other cells than those in suppressive clusters (Figure S8D). Based on this finding, we screened for common ligand–receptor pairs in these cohorts (Figure S8E,F). Some pairs, such as MIF_CD74_CD44, MIF_CD74_CXCR4, MDK_NCL, and APP_CD74, frequently communicated with each other. Using our self‐tested scRNA cohort, we further explored the differences in disulfidptosis activity between patients with BRCA exhibiting brain or liver metastases. We applied the t‐Distributed Stochastic Neighbor Embedding (t‐SNE) approach to reduce the dimensions and colored different cell types and metastatic locations (Figure S8G). Interestingly, the disulfidptosis activity scores were significantly different among malignant cells, myeloid cells, mural cells, endothelial cells, and cancer‐associated fibroblasts (Figure S8H). These findings confirm that disulfidptosis activity is strongly correlated with tumors at the scRNA transcriptome level.

### 2.7 *GYS1* is a potential biomarker for TNBC

**FIGURE 5:**
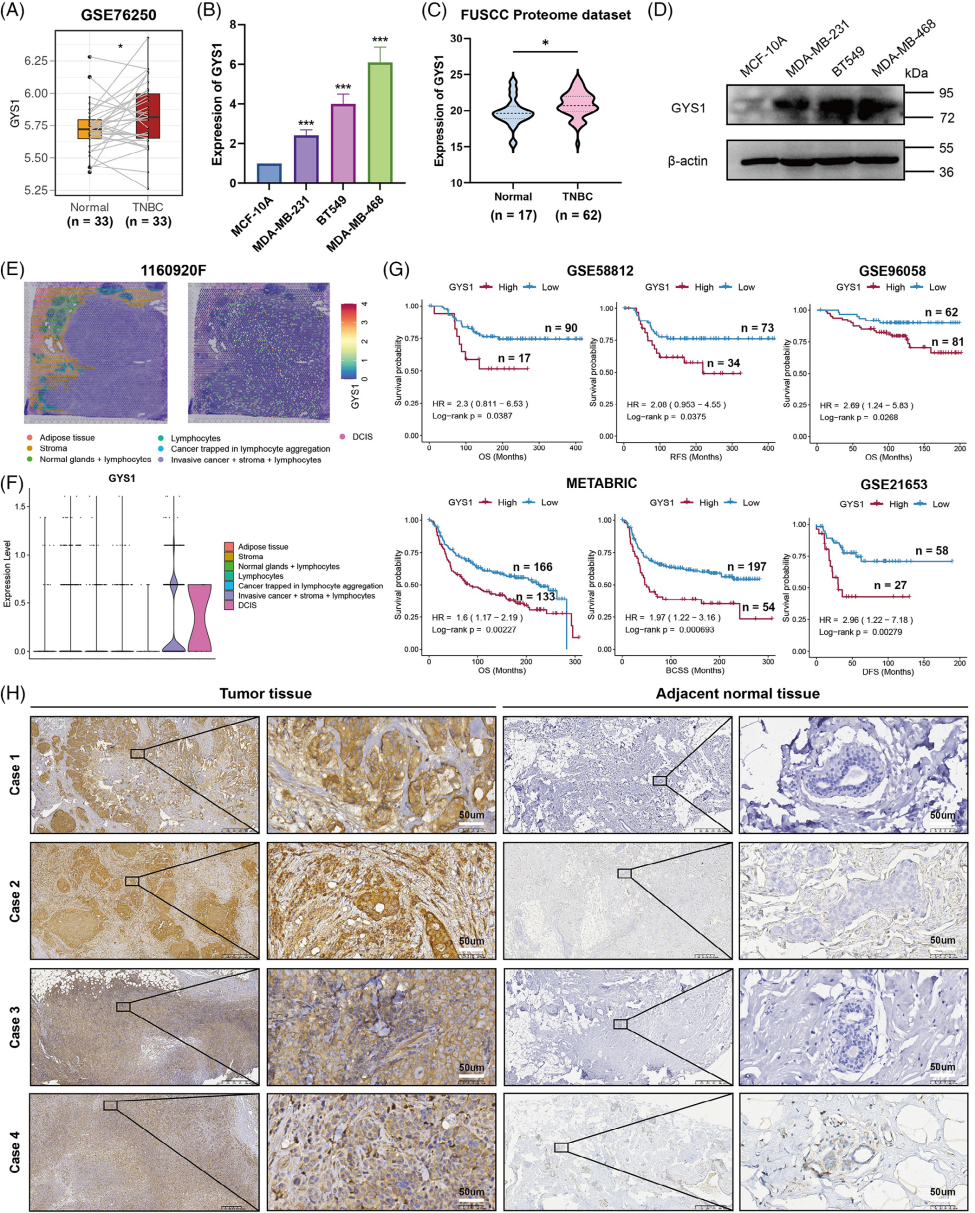
Identification of GYS1 as a potential biomarker in triple‐negative breast cancer (TNBC). (A) Boxplot of the GYS1 expression between 33 paired TNBC and normal tissues. ^*^ *p* < 0.05. (B) Quantitative real‐time polymerase chain reaction (qRT‐PCR) was performed to measure the expression of GYS1 in TNBC cell lines and has been repeated for three times. (C) Violin plot showed the GYS1 expression between TNBC and normal tissues in FUSCC Proteome dataset. ^*^ *p* < 0.05. (D) Western blot assays showing the expression levels of GYS1 in TNBC cell lines. (E) The seven spatial transcriptomic (ST) clusters are overlaid upon the TNBC patient biopsy, and the GYS1 expression levels are also shown. DCIS: ductal carcinoma in situ. (F) Violin plot showed the GYS1 expression level among seven ST clusters. (G) The Kaplan–Meier curve showing the difference in survival time (overall survival [OS], recurrence‐free survival [RFS], breast cancer‐specific survival [BCSS], and disease‐free survival [DFS]) between high and low expression of GYS1. High‐expression GYS1 group is indicated in the red line and low‐expression GYS1 group in blue. (H) Immunohistochemical comparison of GYS1 between paired TNBC (*n* = 4) and adjacent normal tissues (*n* = 4).

Our study indicates that disulfidptosis regulators are crucial for tumor growth and progression. Combined with previous CRISPR/Cas9 of Liu et al., *GYS1* may be the highest‐ranking negative regulator. However, this gene was not explored in detail. TNBC is commonly linked to poor survival outcomes, necessitating the utilization of molecular markers for prognostic and treatment response assessment of BRCA subtypes, and we found that *GYS1* had not been investigated in TNBC. Thus, the expression and clinical relevance of *GYS1* were examined. We collected the public GSE76250 dataset and analyzed the *GYS1* mRNA expression levels in TNBC and paired normal samples. *GYS1* levels were upregulated in the TNBC tissues (Figure 5A). Subsequently, we conducted quantitative real‐time polymerase chain reaction (qRT‐PCR) analysis to validate *GYS1* mRNA expression levels in TNBC cell lines (MDA‐MB‐231, MDA‐MB‐468, and BT‐549). We observed that *GYS1* mRNA expression levels were elevated in TNBC cell lines (Figure 5B), which is consistent with the result of previous bioinformatic analysis results. Besides, we downloaded the FUSCC proteome dataset, which contained proteomic information on TNBC patients, and Figure 5C shows that *GYS1* protein levels were upregulated in TNBC tissues. Western blotting analysis confirmed that *GYS1* protein levels were upregulated in the TNBC cell lines (Figure 5D). A spatial transcriptome (ST) dataset containing TNBC patients was then collected. Figure 5E,F shows that *GYS1* was mainly enriched in the tumor areas such as ductal carcinoma in situ and invasive areas compared with other areas such as stromal and lymphocyte‐infiltrating areas. Furthermore, using GEO datasets (GSE58812, GSE96058, and GSE21653) and METABRIC database, we performed K‐M analyses. Among TNBC patients, higher *GYS1* expression levels were associated with shorter OS, breast cancer‐specific survival, recurrence‐free survival, and DFS (Figure 5G). Moreover, we collected samples from tumors and paired adjacent normal tissues of patients with TNBC to determine the protein levels of *GYS1* via immunohistochemistry (IHC) analysis. As expected, *GYS1* protein levels were significantly higher in TNBC tissues than in the adjacent normal tissues (Figure 5H).

### 2.8 Targeting *GYS1* triggers disulfidptosis via inducing F‐actin contraction in TNBC

**FIGURE 6:**
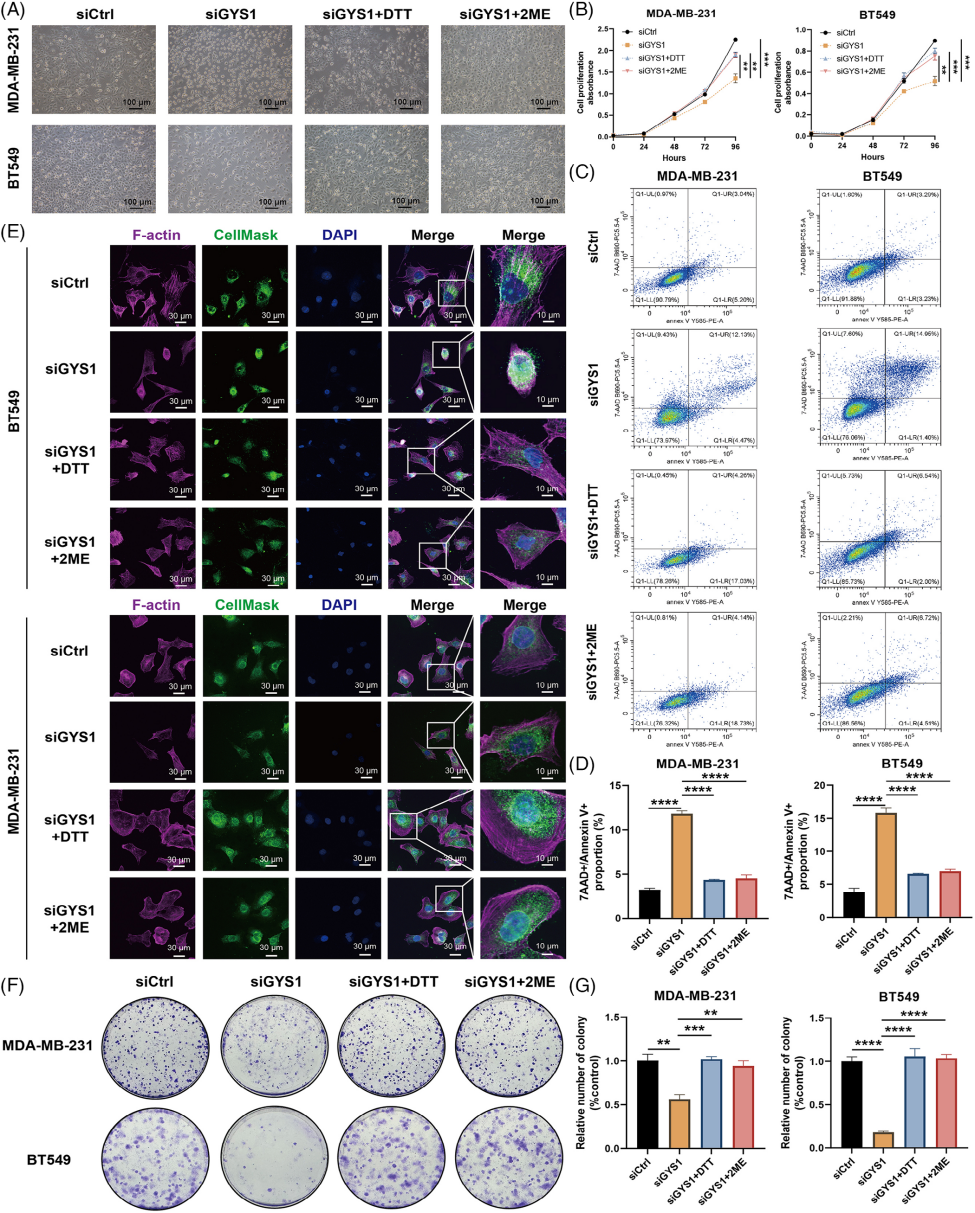
Targeting GYS1 triggers disulfidptosis via inducing F‐actin contraction in triple‐negative breast cancer (TNBC). (A) Representative phase‐contrast images and cell death of TNBC cells treated with siCtrl, siGYS1, siGYS1 + dithiothreitol (DTT), and siGYS1 + β‐mercaptoethanol (2ME). (B) Cell counting kit‐8 (CCK‐8) assays were used for analyzing the growth curves of TNBC cells treated with siCtrl, siGYS1, siGYS1 + DTT, and siGYS1 + 2ME. (C) Flow cytometry was employed to detect the 7‐AAD +/– Annexin V + proportion of TNBC cells treated with siCtrl, siGYS1, siGYS1 + DTT, and siGYS1 + 2ME. (D) Boxplot of the 7‐AAD +/– Annexin V + proportion of TNBC cells treated with siCtrl, siGYS1, siGYS1 + DTT, and siGYS1 + 2ME. ^****^ *p* < 0.0001. (E) Fluorescent staining of F‐actin, membrane (CellMask), and DAPI of TNBC cells treated with siCtrl, siGYS1, siGYS1 + DTT, and siGYS1 + 2ME. (F) Colony formation assays of TNBC cells treated with siCtrl, siGYS1, siGYS1 + DTT, and siGYS1 + 2ME. (G) Boxplot of the relative number of TNBC cell colonies treated with siCtrl, siGYS1, siGYS1 + DTT, and siGYS1 + 2ME. ^**^ *p* < 0.01, ^***^ *p* < 0.001, ^****^ *p* < 0.0001. All above experiments have been repeated for three times.

Next, we explored whether targeting *GYS1* could trigger disulfidptosis in TNBC. MDA‐MB‐231 and BT‐549 were used to explore the biological roles of *GYS1* in TNBC progression. Since *GYS1* was upregulated in the TNBC cell lines, two independent small interfering RNAs (siRNAs) targeting *GYS1* mRNA were generated to efficiently reduce *GYS1* expression in MDA‐MB‐231 and BT‐549 cells (Figure S9). Then, TNBC cells were treated with (a) siCtrl transfection, (b) siGYS1 transfection, (c) siGYS1 transfection + dithiothreitol (DTT), (d) siGYS1 transfection + β‐mercaptoethanol (2ME). DTT and 2ME are effective reducing agents capable of diminishing protein intramolecular or intermolecular disulfide bonds arising from cysteine residues within proteins. We found that TNBC cells transfected with siGYS1 were crumpled compared with other three groups (Figure 6A). Cell counting kit‐8 (CCK‐8) and colony formation assay showed that siGYS1 transfection decreased the tumor cell proliferation, whereas siGYS1 transfection + DTT/2ME maintained the growth of tumor cells (Figure 6B,F,G). Besides, we performed flow cytometry and found that the proportion of 7‐amino actinomycin D (7‐AAD) +/– Annexin V + TNBC cells transfected with siGYS1 was higher than that in the other three groups, indicating that targeting *GYS1* might trigger cell death (Figure 6C,D). Subsequently, we performed fluorescent staining with F‐actin, CellMask (cell membrane), and 4,6‐diamidino2‐phenyl‐indole dihydrochlordie (DAPI) and observed F‐actin contraction in TNBC cells transfected with siGYS1 (Figure 6E). These findings suggest that targeting *GYS1* might trigger disulfidptosis via inducing F‐actin contraction in TNBC.

### 2.9 In vitro and in vivo experimental validations of *GYS1* in TNBC

**FIGURE 7:**
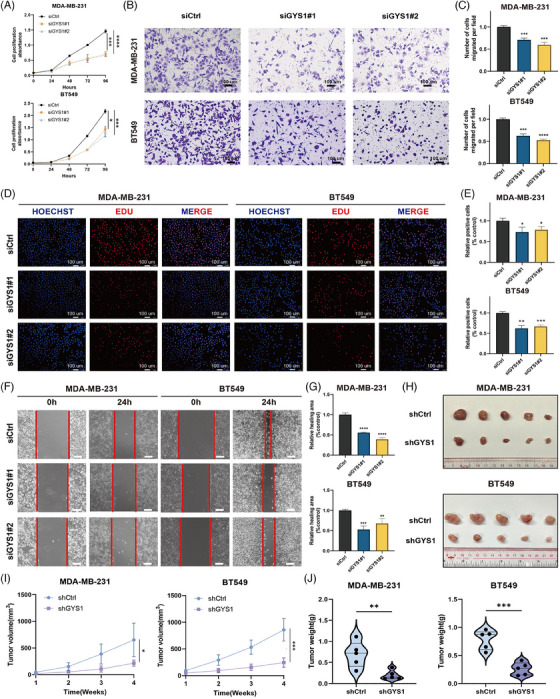
In vitro and in vivo validations of GYS1 as a potential biomarker in triple‐negative breast cancer (TNBC). (A) Cell counting kit‐8 (CCK‐8) assays were used for analyzing the growth curves of TNBC cells and have been repeated for three times. (B) Transwell migration assays (scale bar = 100 μm) were performed to measure the migration abilities in TNBC cells and have been repeated for three times. (C) Boxplots of the number of cells migrated per field in TNBC cells. ^***^ *p* < 0.001, ^****^ *p* < 0.0001. (D) EdU assays was conducted to evaluate proliferation of TNBC cells (scale bar = 100 μm) and have been repeated for three times. (E) Boxplots of the relative positive cells in EdU assays. ^*^ *p* < 0.05, ^**^ *p* < 0.01, ^***^ *p* < 0.001. (F) Wound healing assays (scale bar = 100 μm) were performed to measure the migration abilities in TNBC cells and have been repeated for three times. (G) Boxplots of relative healing area (% control) in TNBC cells. ^**^ *p* < 0.01, ^***^ *p* < 0.001, ^****^ *p* < 0.0001. (H) The images of xenograft tumor in control group and shGYS1 group were displayed (*n* = 5). (I) The tumor volumes were measured once a week and the growth curves of each group were drawn. ^**^ *p* < 0.01, ^***^ *p* < 0.001. (J) The tumor weights of each group were measured and analyzed. ^**^ *p* < 0.01, ^***^ *p* < 0.001.

Next, we performed in vitro and in vivo experimental validations of *GYS1* in TNBC cell lines. CCK‐8 and 5‐Ethynyl‐2'‐deoxyuridine (EdU) assays were conducted to estimate the effects of *GYS1* on TNBC cells viability. Knockdown of *GYS1* significantly decreased the viability of MDA‐MB‐231 and BT‐549 cells (Figure 7A,D,E). Moreover, we performed Transwell and wound healing assays to confirm whether *GYS1* is correlated with the migratory capacity of TNBC cells. Notably, *GYS1* knockdown significantly attenuated TNBC cells migration (Figure 7B,C,F,G).

Next, xenograft models were created utilizing MDA‐MB‐231 and BT‐549 cells that underwent stable *GYS1* knockdown and their corresponding control cells (Figures 7H and S10). We found that the *GYS1* knockdown group exhibited a statistically significant reduction in both tumor weight and volume compared with the control group (Figure 7I,J).

In summary, these results showed that *GYS1* promotes TNBC cells proliferation and migration in vitro and in vivo, suggesting its role as a potential biomarker for TNBC.

## 3 DISCUSSION

The modification of metabolic processes is a distinguishing characteristic of cancer and offers an opportunity for targeted intervention in cancer therapy.^40^ Actin networks can collapse if the disulfide bonds among proteins are not repaired, leading to cell death. Disulfidptosis, a novel type of cell death, has been excavated recently, and disulfidptosis regulators are screened, which will ultimately contribute to the advancement of therapeutic approaches targeting cancer.^41^ It is crucial to recognize the underlying mechanisms of disulfidptosis, as they have significant impacts on understanding the fundamentals of cellular homeostasis, as well as providing insight into treatment of diverse diseases such as cancer.

Using multi‐omics pan‐cancer cohorts, our study offers a comprehensive analysis of the molecular and clinical characteristics of disulfidptosis regulators. We investigated the expression and prognosis patterns of disulfidptosis regulators and found that these regulators exhibited significant heterogeneity in expression as well as in terms of prognosis, which necessitates the study of each regulator in specific tumor type. Growing evidence has confirmed that genetic mutations play vital roles in tumorigenesis and progression.^42^ We found that *CYFIP1* and *LRPPRC* had the highest SNV frequencies among these disulfidptosis regulators (15%). *CYFIP1* is a component of the well‐known WASF regulatory complex, along with *NCKAP1*, *ABI1*, and *BRK1*.^43^ *LRPPRC* belongs to the pentatricopeptide repeat family. Previous studies verified that *LRPPRC* mutation might alter the stability of mitochondrial RNA transcripts, thereby suppressing cytochrome oxidase activity.^44^, ^45^ In accordance with our expectations, the expression levels of disulfidptosis regulators were positively related to CNV profiles in most tumors, indicating that tumors with CNV might exhibit changes in their corresponding expression. As for methylation analyses, we observed that the methylation levels of *SLC7A11* were consistently decreased in tumor samples, suggesting that hypomethylation might induce the transcriptional activation of *SLC7A11* which results in disulfidptosis.

Considering the crucial role of disulfidptosis, we constructed a disulfidptosis activity score and validated it using multiple datasets. We found that tumor cells under glucose starvation exhibited significantly higher disulfidptosis activity scores, whereas those under high glucose treatment displayed lower disulfidptosis activity scores. Besides, a previous study found that disulfidptosis is a novel way to induce cell death, which is not affected by other type of inhibitors, and we found that ferroptosis inhibitors and autophagy inhibitors did not make impacts on disulfidptosis activity scores. Pathway activity analyses revealed a strong association between disulfidptosis and oxidative phosphorylation. Additionally, the disulfidptosis activity score was positively related to mitotic spindle, transforming growth factor‐beta (TGF‐β) pathways, Wnt‐β‐catenin pathways, etc., confirming its crucial role in tumor growth and metastasis and directly giving a theoretical basis for further research. Moreover, our study revealed that disulfidptosis was also related to immune‐related characteristics, and patients with high‐disulfidptosis activity obtained better prognoses after immunotherapy than those with low‐disulfidptosis activity, indicating that the disulfidptosis activity score might be predictive of immunotherapy efficacy in tumor patients. Furthermore, disulfidptosis activity also showed tight correlations with tumors at scRNA‐seq, and pairs, such as MIF_CD74_CD44, MIF_CD74_CXCR4, MDK_NCL, and APP_CD74, might be potential targets in disulfidptosis‐related tumor microenvironment.

Combined with the existing result from CRISPR‐Cas9 screening in Liu et al. study, *GYS1* possessed the highest ranking among the negative regulators. However, they did not explore this gene deeply. Another study found that targeting *GYS1* exposed a metabolic weakness in TNBC.^46^ Nevertheless, they mainly focus on glycogen metabolism‐related representations and lack multi‐omics validations. Besides, how *GYS1* deletion leads to TNBC cells death remains unclear. Here, we conducted integrated bioinformatics and experiments analyses of *GYS1* in TNBC. We focused the biological and clinical importance of *GYS1* in TNBC. Strikingly, the result showed that targeting *GYS1* might trigger disulfidptosis via inducing F‐actin contraction in TNBC. Besides, *GYS1* promoted TNBC cells proliferation and migration in vitro and in vivo experimental validations, suggesting it might be a potential biomarker for TNBC. Several observations have indicated that there is a modification in the turnover of glycogen within tumor cells, and studies revealed that breast, kidney, uterus, bladder, ovary, skin, and brain cancer cell lines exhibited notably elevated levels of glycogen.^47^ Thus, we hypothesized that *GYS1* might promote cellular glycogen synthesis and accumulation, which provides the raw material for tumor cells to maintain a high level of the NADPH pool, thus providing critical reducing capacity to counteract disulfide stress and maintain cell survival.

Notably, our study still remains some limitations. First, our results are widely based on big‐data analyses, which limits our study. Therefore, further experiments using novel technologies, such as metabolomics, are necessary to verify our findings and determine the underlying mechanisms. Second, further verifications are still required to determine how *GYS1* can be translated into clinical treatment for TNBC patients. A phase I clinical trial (NCT05249621) is currently underway to assess the safety and determine the maximum tolerated dose of a newly developed *GYS1*‐specific inhibitor. This inhibitor exhibits potent inhibitory effects on *GYS1* in cell lysates derived from non‐cancerous cells. Therefore, in vivo experiments such as patient‐derived tumor xenograft models need to be conducted, and the combination of *GYS1* inhibitors with classic anti‐tumor drugs is imperative to be explored in forthcoming studies.

## 4 CONCLUSION

Overall, our study offers a pan‐cancer blueprint of the molecular and clinical characteristics of disulfidptosis regulators and disulfidptosis activity in tumors using multi‐omics pan‐cancer cohorts, providing a new direction for future research.

## 5 MATERIALS AND METHODS

### 5.1 Data collection

Normal tissue expression data from the GTEx dataset and the gene expression data from TCGA pan‐cancer dataset were obtained from UCSC Xena website. Corresponding clinical information for each sample was downloaded. The sample sizes of different tumor types are summarized in Table S1. We constructed an interaction network of each disulfidptosis regulator using the STRING database, and the results are shown using the “circlize” R package.^48^ Mutation repositories (SNV and CNV) and methylation levels of disulfidptosis regulators were obtained from Gene Set Cancer Analysis website. Only samples containing more than two normal samples were considered for mRNA differential expression analysis. For methylation analysis, only those possessing a minimum of five tumor‐normal pairs were selected. We downloaded 50 hallmark pathways from MSigDB website. Classic immune checkpoints and immunotherapy‐predicted pathways have been reported in previous studies.^49^, ^50^ Multiple existing microenvironmental deconvolution methodologies were applied with “IOBR” R package.^51^ The cancer immunity cycle of TCGA pan‐cancer patients was obtained from the TIP website.

Bulk RNA‐seq data from GSE62663,^52^ GSE121378,^53^ GSE183127,^54^ GSE194369, GSE104462,^55^ GSE32369,^56^ GSE206261,^57^ GSE171167, GSE144833,^58^ CheckMat,^59^ IMvigor210,^60^ GSE91061,^61^ GSE78220,^62^ GSE58812,^63^ GSE96058,^64^ METABRIC, GSE21653,^65^ and GSE76250^66^ were extracted for independent analysis. Probes were mapped using “AnnoProbe” R package. Average values of multiple probes were calculated by the “limma” R package.^67^ We also collected scRNA‐seq data from GSE117570,^68^ GSE160269,^69^ EMTAB8107,^70^ GSE176078,^71^ and from our previous study.^72^ Proteome dataset was obtained from a previous study.^73^ ST data of a TNBC patient was obtained from Zenodo data Repository (https://doi.org/10.5281/zenodo.3957257). We also collected 21 disulfidptosis regulators from published articles, and their relationships were determined using the STRING database.^1^, ^12^ All datasets enrolled in this study are summarized in Table S2.

### 5.2 Survival analysis

We conducted K‐M and Cox regression analyses using R packages “survminer” and “survival.” “surv_cutpoint” function was applied to calculate the cutoff values. We also determined 95% confidence intervals and hazard ratios.

### 5.3 Pathway activity analyses

We applied the ssGSEA algorithm (“GSVA” R package) to assess the enrichment level of pathway activity in each sample.^74^ Disulfidptosis‐positive score was based on the positive disulfidptosis regulators and the disulfidptosis‐negative score was based on the negative disulfidptosis regulators, and the disulfidptosis activity score was defined as the difference between them.

### 5.4 scRNA‐seq and ST data processing

Annotated cell types were all obtained from previous studies. “Seurat” R package was used for subsequent analyses.^75^ All functions were executed using their default parameters, unless explicitly specified otherwise. For ST data, cells annotated with “artifact” were filtered. We used t‐SNE and UMAP to reduce the dimensions and classify each cell type. The disulfidptosis activity score of each cell was calculated by “AddModulScore” function. Cell–cell communication analysis between immune/stromal and epithelial cells was performed by “CellChat” R package.^76^

### 5.5 Human BC cell lines and cell culture

Human epithelial BC cell lines (MCF10A, MDA‐MB‐231, BT549, and MDA‐MB‐468) were obtained from the American Type Culture Collection. Standard guidelines were followed to culture all cell lines and maintain them at 37°C and 99% relative humidity without antibiotics. siRNA oligos against *GYS1* were transfected using Lipofectamine 3000 (Invitrogen). All sequences of siRNAs used in this study are listed in Table S3. TNBC cells were transfected with the shRNA‐GYS1 or control viruses and the sequences are listed in Table S4.

### 5.6 RNA isolation and qRT‐PCR analysis

Total RNA was extracted from cells using the RNA‐Quick Purification Kit (ES‐RN001, Shanghai Yishan Biotechnology Co.). Table S5 shows the primer sequences. We performed qRT‐PCR to determine the RNA levels on a Bio‐Rad CFX96 using the SYBR Green method (RR420A; Takara). The qRT‐PCR plates were purchased from NEST (402301; Wuxi NEST Biotechnology Co.). Comparative Ct method was used to normalize the RNA levels against β‐actin RNA.

### 5.7 Western blotting analysis

Protein extracts from cells were prepared using RIPA lysis buffer. Total proteins were separated via SDS‐PAGE and transferred to PVDF membrane (Millipore). Antibodies against *GYS1* and β‐actin were used. Membranes were incubated with the primary antibody at 4°C overnight and then with secondary antibody at room temperature for 1 h. The blots were further visualized with Immobilon Western Chemiluminescent horseradish peroxidase (HRP) Substrate (Beyotime).

### 5.8 Tissue collection and IHC staining

Tumor and adjacent mammary tissues of TNBC patients utilized for IHC were retrospectively obtained from the Sun Yat‐Sen University Cancer Center, with prior informed consent obtained from each patient. Tissue‐containing sections were deparaffinized with xylene and subsequently rehydrated using a series of graded ethanol dilutions (100%, 95%, 85%, and 75%). Prior to overnight incubation with the primary antibody at 4°C, endogenous peroxidase activity was blocked and antigen retrieval was conducted. Subsequent to a 20‐min incubation with an HRP‐conjugated secondary antibody at ambient temperature, staining was performed utilizing diaminobenzidine (DAB) substrate (Dako). Following DAB treatment, sections were stained with hematoxylin.

### 5.9 Flow cytometry analysis

Cell death was analyzed via flow cytometry. TNBC cells were washed twice with phosphate‐buffered saline and subsequently resuspended in 100 μL of 1× binding buffer. This suspension was combined with 2.5 μL of Annexin V‐PE and 2.5 μL of 7‐AAD staining solution, and incubated for 15 min in the absence of light at room temperature. Following this, 400 μL of additional binding buffer was added into the mixture, and the cells were ultimately assessed using a flow cytometer.

### 5.10 Fluorescent staining of actin filaments and cellular membrane

Immunofluorescence was conducted as previously described.^72^ Briefly after 10 h of treatment, the cells were washed twice with phosphate‐buffered saline (PBS) and fixed with 3.7% paraformaldehyde for 20 min at room temperature. For actin filament staining, the fixed cells were permeabilized for 5 min at room temperature with 0.1% Triton X‐100 in PBS twice. The cells were incubated at room temperature in the dark for 45 min with Actin‐Tracker Red‐555 phalloidin (#C2203S; Beyotime). Next, the cells were washed for 5 min at room temperature with 0.1% Triton X‐100 in PBS twice. For co‐staining of actin filaments and cellular membrane the above‐mentioned fixed cells were incubated at 4°C in the dark for 30 min with PKH67 CellMask Green Plasma Membrane Stain dye (#D0031; Solarbio) and washed for 5 min at room temperature with in PBS twice. Afterwards, cell nuclei were stained by DAPI (#BL105A; Biosharp) at 37°C for 15 min. All fluorescence images were captured using a confocal microscope (FV 1000, OLYMPUS).

### 5.11 Colony formation assays

TNBC cells were inoculated in six‐well plates at a density of 2500 cells per well and treated with 0.2 mM DTT/1 mM 2ME after 48 h. After 12 days, the colonies were fixed using methanol and subsequently stained with crystal violet solution (0.1%), imaged and counted.

### 5.12 CCK‐8 assays

A total of 2000 cells were incubated in 96‐well plates. Subsequently, the CCK‐8 solution was introduced into each well and incubated for a duration of 2 h for evaluating cell proliferation. The measurement of absorbance at OD450 was conducted from days 0 to 4.

### 5.13 EdU assays

TNBC cells were seeded on coverslips in 12‐well plates incubated under standard culture conditions. After 24 h, EdU was introduced into the culture medium and subsequently incubated at a temperature of 37°C for a duration of 3 h. Next, the sample was treated with a 4% solution of paraformaldehyde for a duration of 20 min at ambient temperature. Following the removal of paraformaldehyde, a solution containing 0.3% Triton X‐100 (Invitrogen) was introduced into the plates and allowed to incubate for a duration of 20 min. Subsequently, the Click Additive Solution was incorporated following the guidelines outlined in the commercial protocol (BeyoClick EdU‐555), and subsequently incubated at ambient temperature for a duration of 30 min, while being shielded from light. Finally, the nucleus was stained with Hoechst 33342 and images were captured using a fluorescent microscope manufactured by Olympus.

### 5.14 Transwell assays

A total of 60,000 cells were subjected to digestion and subsequently resuspended. Cells from each experimental group were introduced into the upper chambers, which were devoid of fetal bovine serum (FBS), while the lower cross‐pore compartment contained a solution with 20% FBS. Following a 22 h period, we conducted imaging and quantification of all migrated TNBC cells subsequent to their fixation with methanol and staining with crystal violet (0.1%).

### 5.15 Cell wound healing assays

The TNBC cells underwent transfection were cultured in 6‐well plates at a density of 1 × 10^6^ cells per well for a duration of 24 h. Next, the wounds were generated utilizing a 100 μL pipette tip. The images were captured using a microscope at both 0 and 24 h. We employed image J software to measure the scratch area and assess cell migratory capacity.

### 5.16 Animal experiments

BALB/c female nude mice aged four weeks were procured from Zhuhai BesTest Bio‐Tech Co. Ltd. and were subsequently housed at the Animal Facility of Sun Yat‐Sen University Cancer Center under controlled conditions. Nude mice were subcutaneously injected with 2 × 10^6^ PBS suspended MDA‐MB‐231 and BT‐549 cells with stable *GYS1* knockdown or control cells, and were sacrificed after 4 weeks.

### 5.17 Statistical analysis

We used R version 4.2.0 to perform all analyses. The fold change was determined by dividing the mean expression of the tumor sample by the mean expression of the normal sample. We used Wilcoxon rank‐sum test to compare the differences between two groups and Kruskal–Wallis test to three or more groups. Spearman correlation coefficient was chosen to accomplish correlation analysis. The false discovery rate (FDR) was calculated with the Benjamini–Hochberg adjustment method. *p* < 0.05 or FDR < 0.05 was considered statistically significant.

## AUTHOR CONTRIBUTIONS

*Research design*: Y.Z., X.X., and M.C. *Data collection*: J.X., X.D., Y.X., and H.Z. *Data analysis*: J.X., X.D., Y.X., H.Z., P.L., and W.D. *Manuscript preparation*: J.X., X.D., Y.X., H.Z., P.L., W.D., L.N., Y.T., Y.S., and H.T. *Manuscript editing*: Y.Z., X.X., and M.C. All authors have read and approved the final manuscript

## CONFLICT OF INTEREST STATEMENT

The authors declare they have no conflicts of interest.

## ETHICS STATEMENT

Sun Yat‐Sen University Cancer Center's Institute Research Ethics Committee approved this study. All animal experiments were conducted in accordance with protocols approved by the Sun Yat‐Sen University Cancer Center Animal Care and Use Committee (approval ID: SL‐G2023‐294‐01). The tumor specimens and the adjacent mammary tissues utilized for immunohistochemistry in TNBC patients were obtained retrospectively from the Sun Yat‐Sen University Cancer Center (approval ID: GZR‐2021‐358).

## Supporting information

Supporting Information

## Data Availability

All datasets involved in this study can be viewed in the UCSC Xena database (https://xenabrowser.net/datapages/), the Molecular Signature Database (MSigDB) (https://www.gsea‐msigdb.org/gsea/msigdb/), Gene Expression Omnibus, Gene Set Cancer Analysis (http://bioinfo.life.hust.edu.cn/GSCA/), STRING database (https://string‐db.org/), TIP (http://biocc.hrbmu.edu.cn/TIP/index.jsp), or data availability part of the corresponding articles. All data pertinent to this study, whether generated or analyzed, are comprehensively presented in this manuscript and its supplementary information. For any additional inquiries or requests, interested parties are encouraged to contact the corresponding authors.
