# Supplementary material for: Multi‐omics analysis of disulfidptosis regulators and therapeutic potential reveals glycogen synthase 1 as a disulfidptosis triggering target for triple‐negative breast cancer
Source: MedComm (2020). 2024 Feb 28;5(3):e502. doi: 10.1002/mco2.502 (PMC10901283; doi:10.1002/mco2.502)
Supplement: Supplementary file 1 — Supporting Information [file MCO2-5-e502-s001.pdf]

Multi-omics analysis of disulfidptosis regulators and therapeutic potential reveals glycogen synthase 1 as a disulfidptosis triggering target for triple-negative breast cancer

Jindong Xie[1, #], Xinpei Deng[1, #], Yi Xie[1, #], Hongbo Zhu[2], Peng Liu[1], Wei Deng[1], Li Ning[1], Yuhui Tang[1], Yuying Sun[1], Hailin Tang[1], Manbo Cai[2, *], Xiaoming Xie[1, *], Yutian Zou[1, *]

[1] State Key Laboratory of Oncology in South China, Guangdong Provincial Clinical Research Center for Cancer, Sun Yat-sen University Cancer Center, Guangzhou, 510060, China.

[2] The First Affiliated Hospital, Hengyang Medical School, University of South China, Hengyang, Hunan, 421001, China.

*** Correspondence:**

Yutian Zou (zouyt@sysucc.org.cn); Xiaoming Xie (xiexm@sysucc.org.cn):

State Key Laboratory of Oncology in South China, Guangdong Provincial Clinical Research Center for Cancer, Sun Yat-sen University Cancer Center, 651 Dongfeng East Road, Guangzhou, 510060, China.

Manbo Cai (caimanbo@nhfyyy.com):

The First Affiliated Hospital, Hengyang Medical School, University of South China, Hengyang, Hunan, 421001, China.

\# These authors contributed equally to this work.

Supplementary Figure Legends

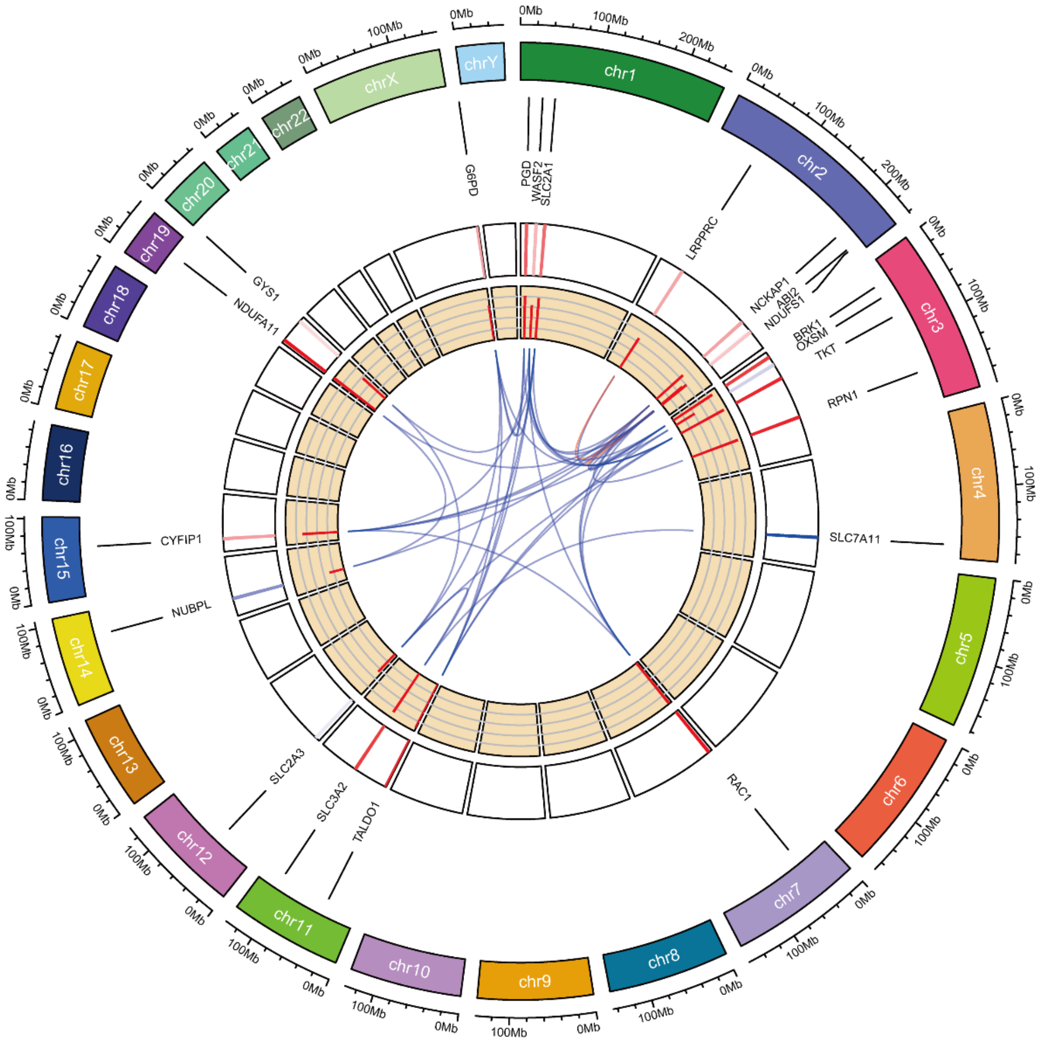

Supplementary Figure S1. Circos plot showed interaction relationships among ten disulfidptosis regulators.

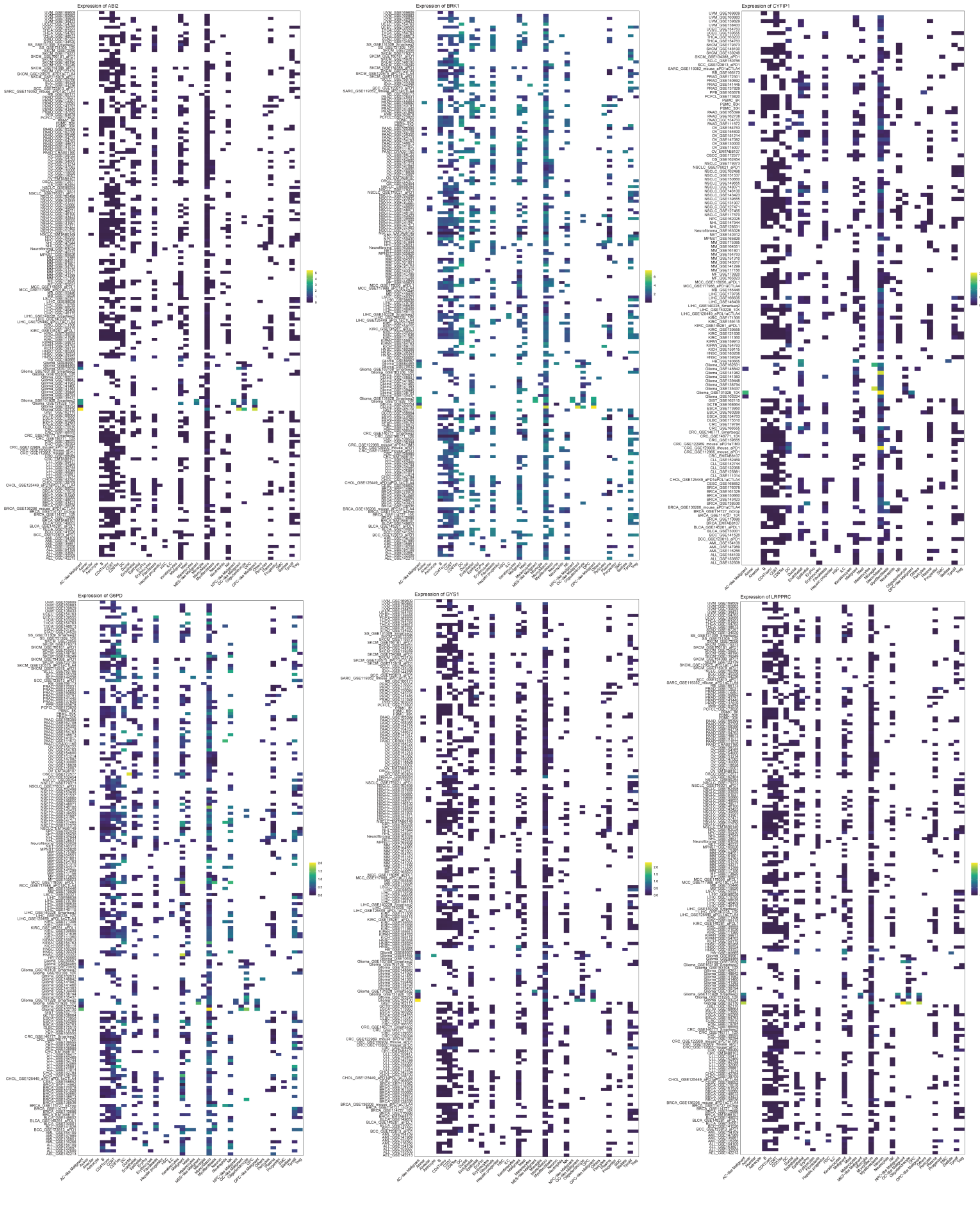
Expression of ABI2
Expression of BRK1
Expression of CYFIP1
Expression of G6PD
Expression of GYS1
Expression of LRPPRC

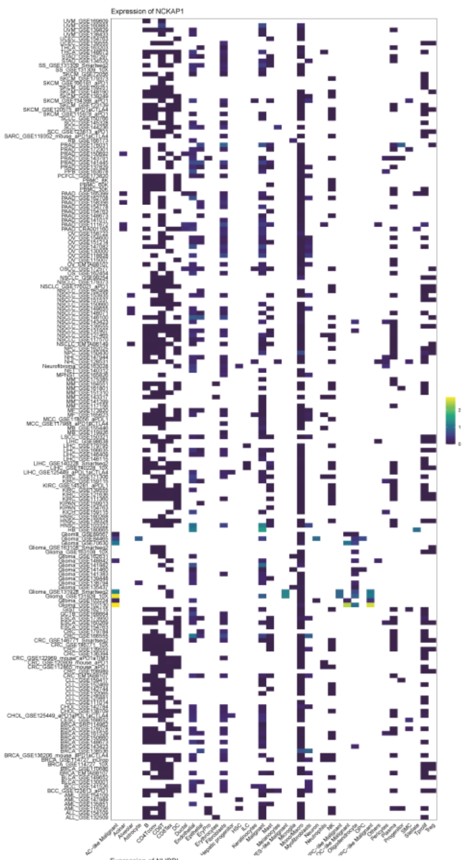
Expression of NCKAP1

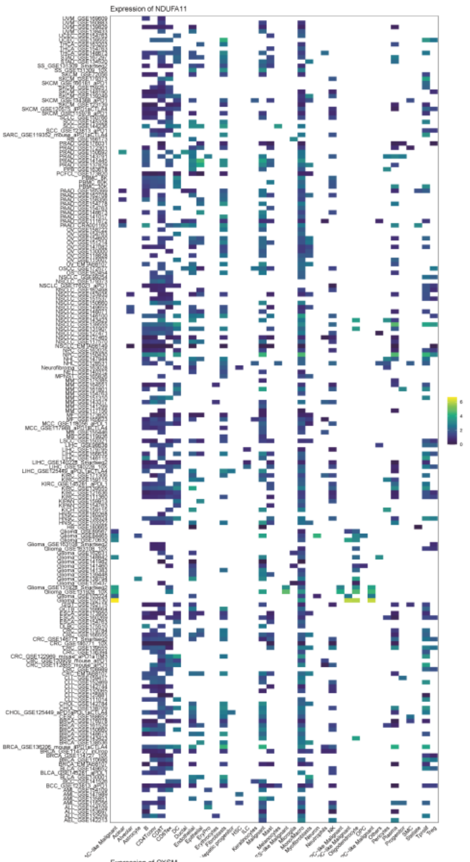
Expression of NDUFA11

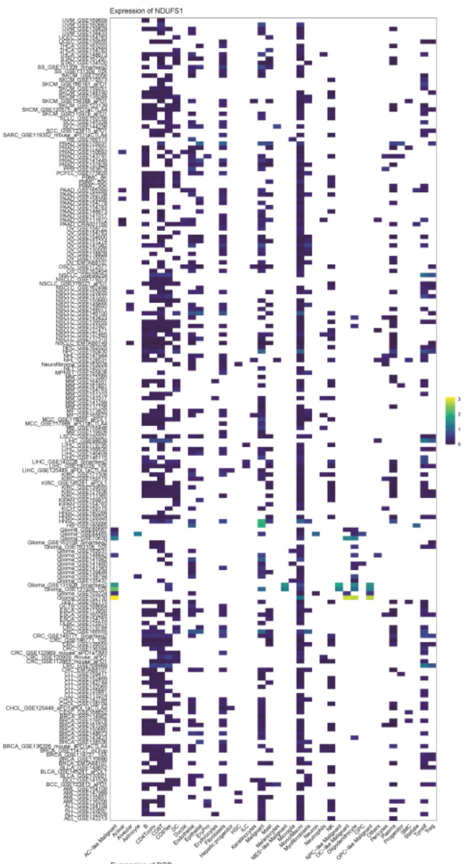
Expression of NDUFS1

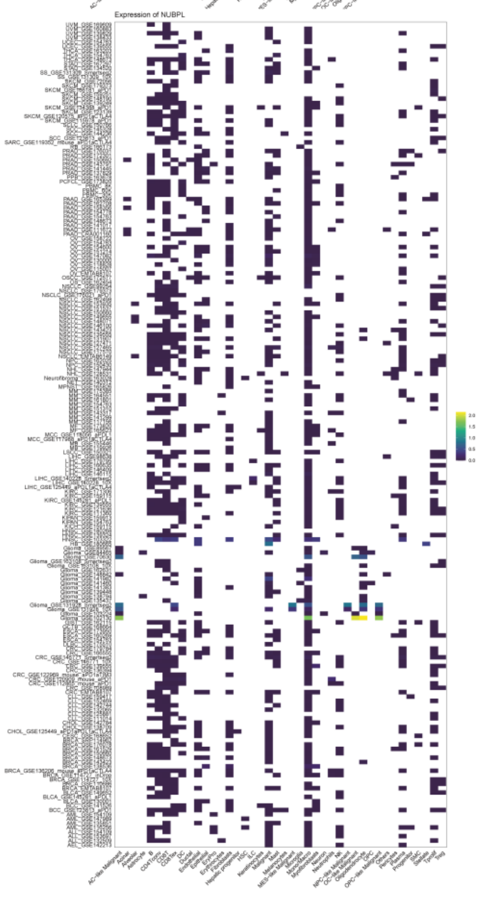
Expression of NUBPL

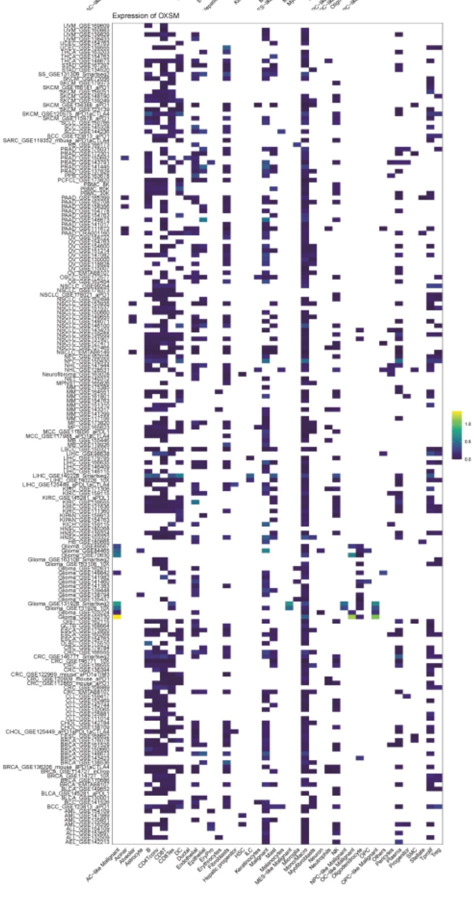
Expression of OXSM

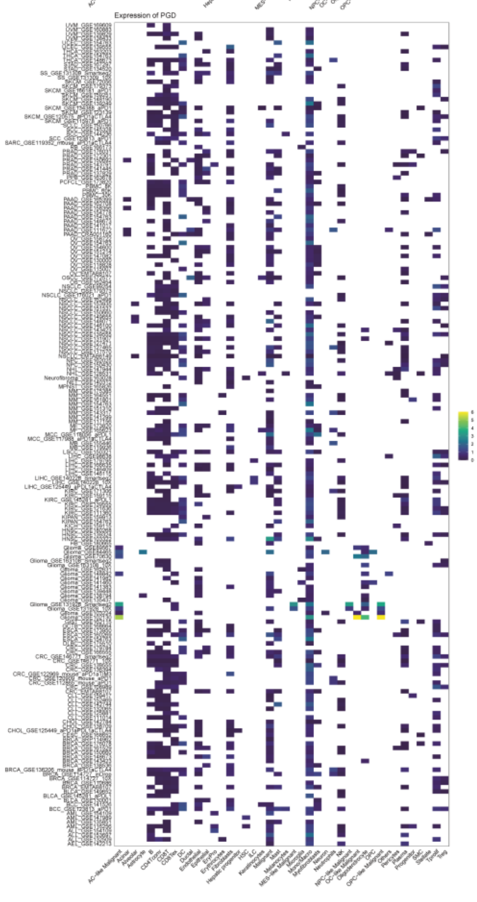
Expression of PGD

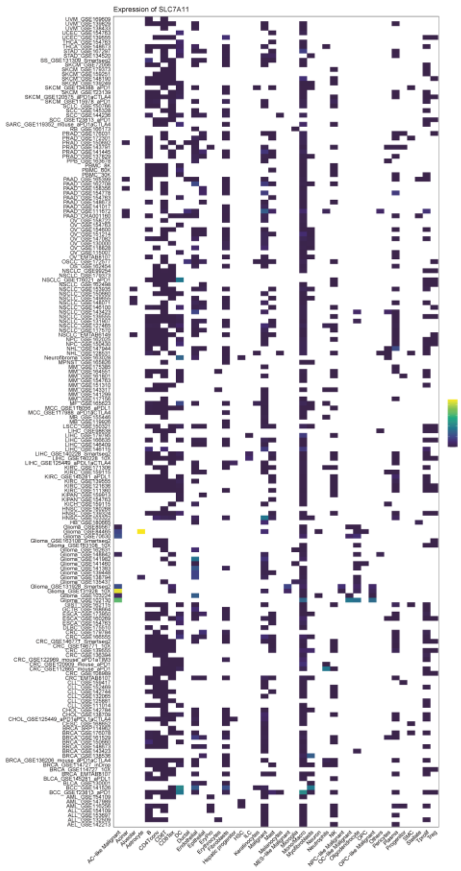
Expression of SLC7A11

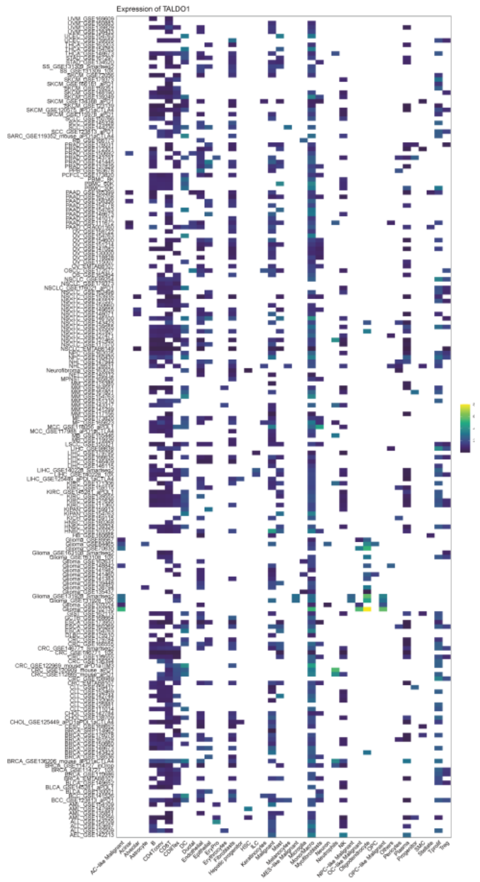
Expression of TALDO1

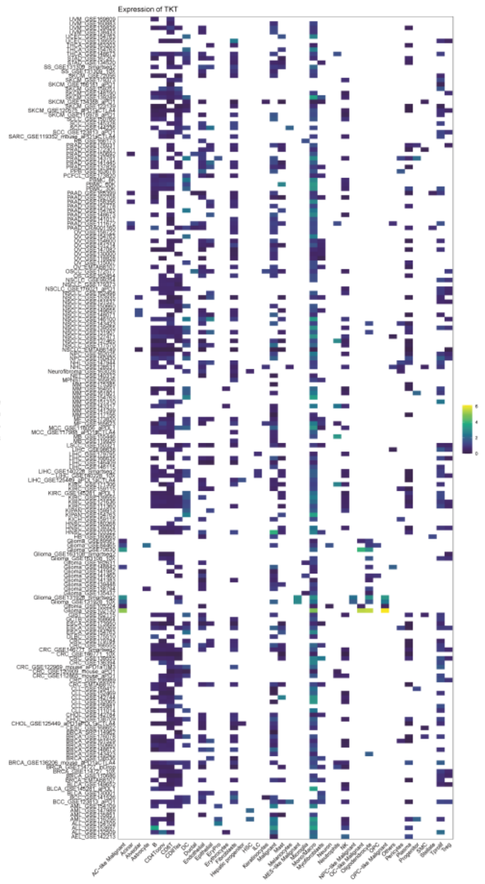
Expression of TKT

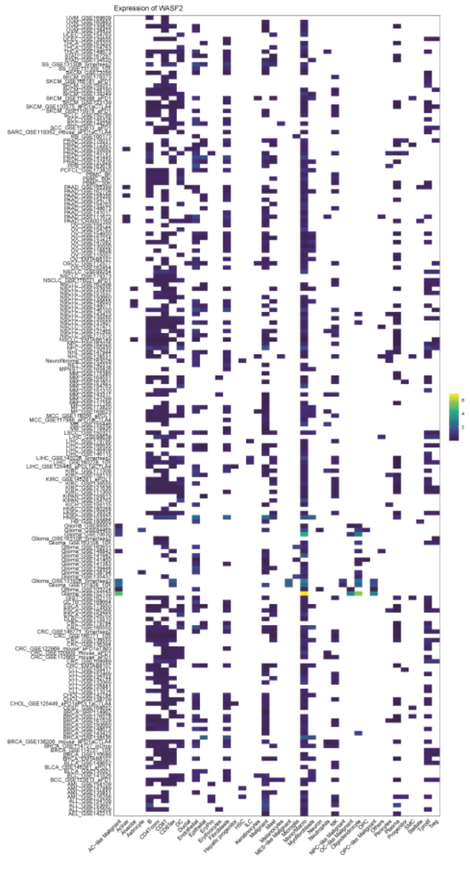
Expression of WASF2

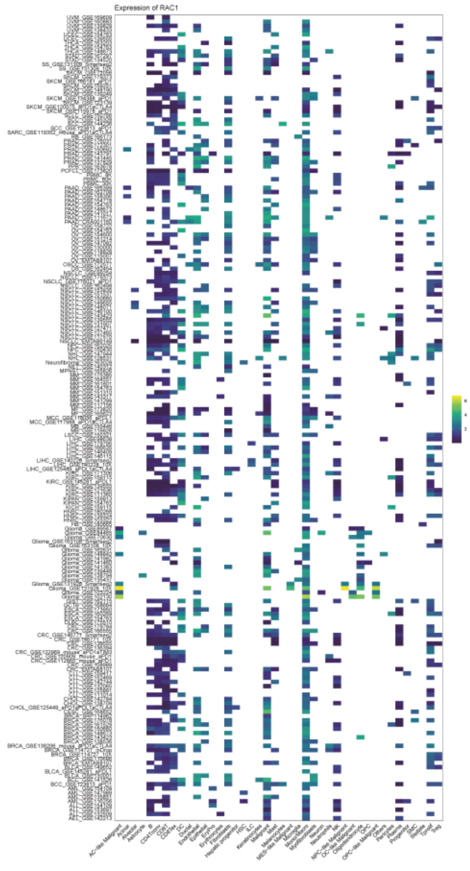
Expression of RAC1

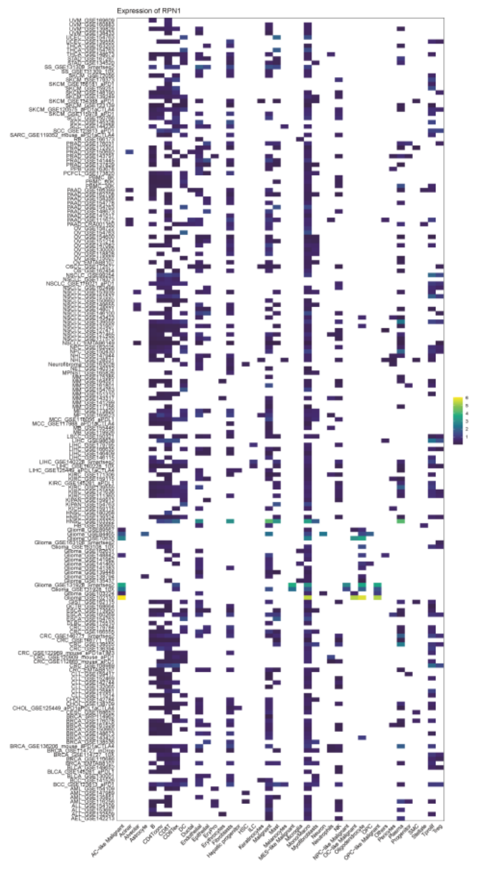
Expression of RPN1

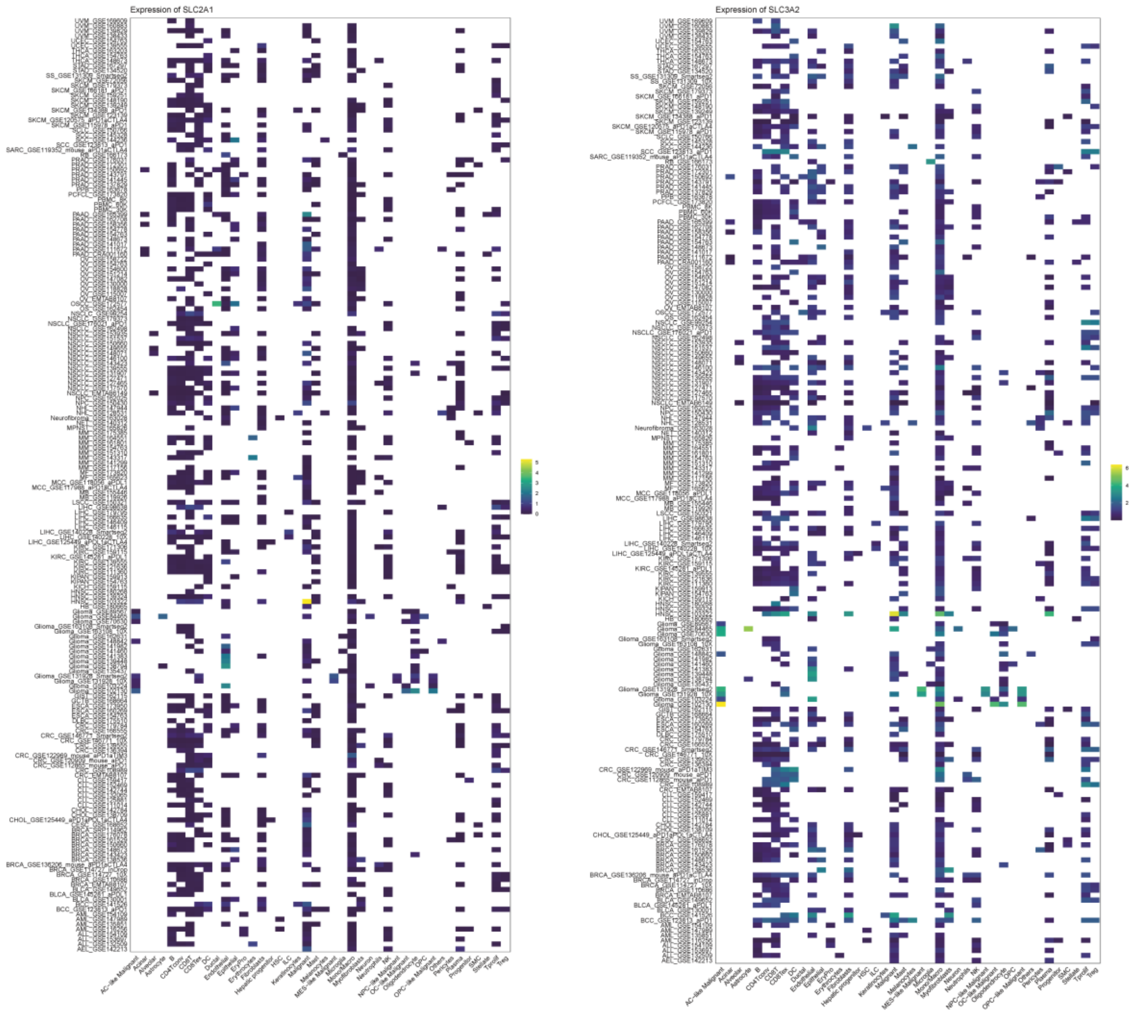

Supplementary Figure S2. The expression and distribution of each disulfidptosis regulator using TISCH database.

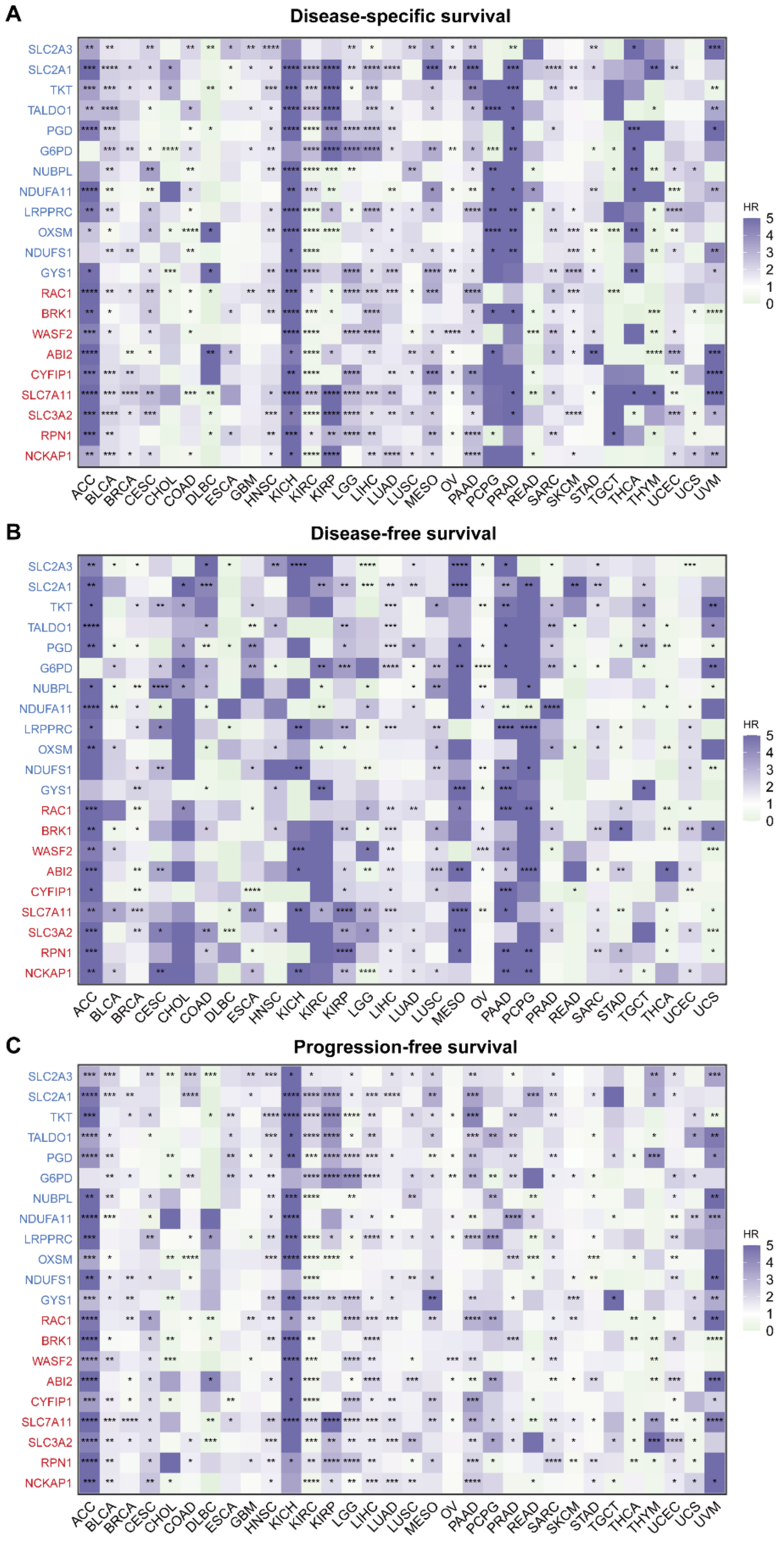

Supplementary Figure S3. Prognosis patterns of disulfidptosis regulators in the TCGA pan-cancer cohorts. Risky and protective genes are marked in purple and green, respectively (HR, hazard ratio; * p < 0.05, ** p < 0.01, *** p < 0.001, **** p < 0.0001).

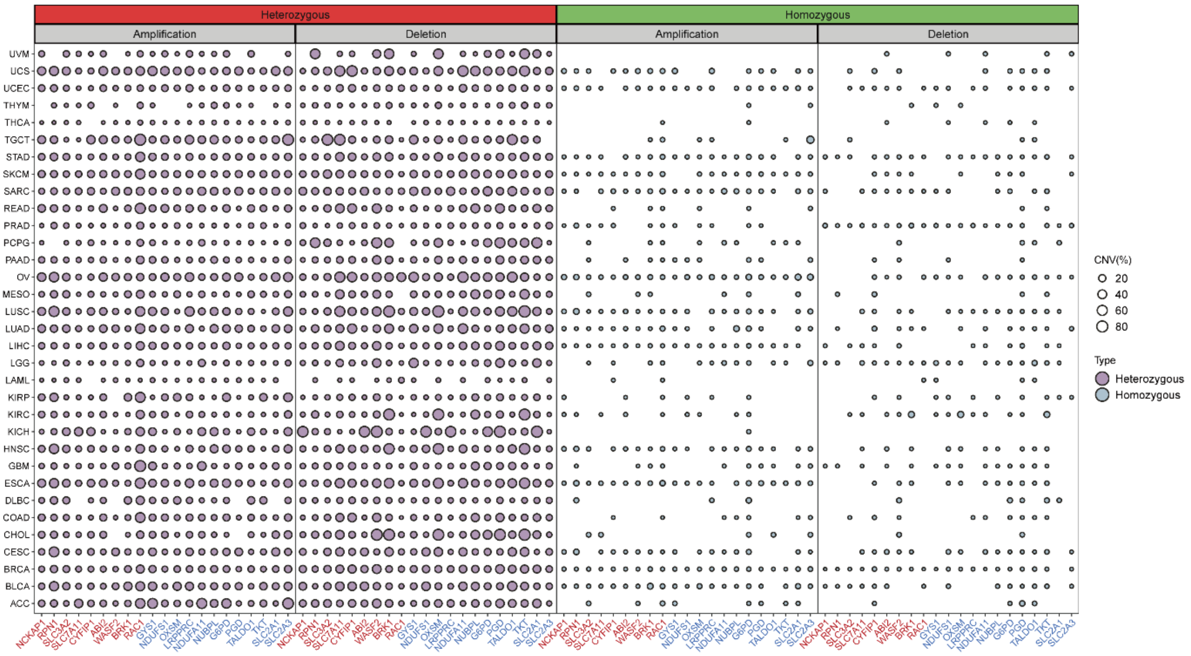

Supplementary Figure S4. The heterozygous and homozygous copy number variation (CNV) profile of disulfidptosis regulators in each tumor type, including the percentage of amplification and deletion.

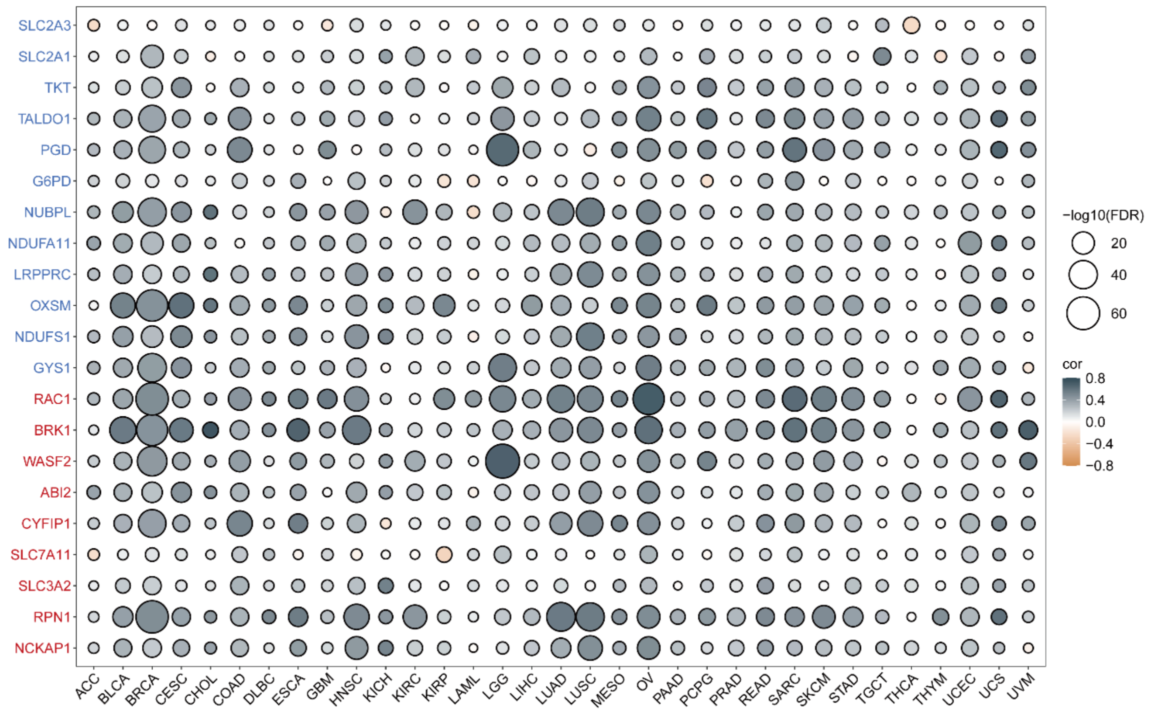

Supplementary Figure S5. Bubble plot of the correlations between CNV profiles and each disulfidptosis regulator expression in pan-cancer (FDR, false discovery rate).

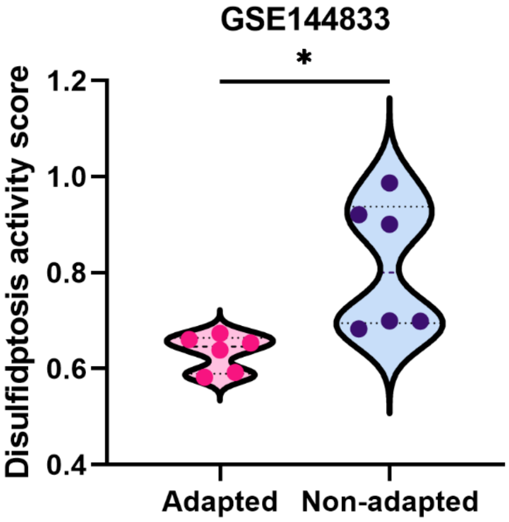

Supplementary Figure S6. Violin plot showed the different disulfidptosis activity scores between pancreatic cancer cells adapted (n=6) or non-adapted (n=6) to nutrient deprivation.

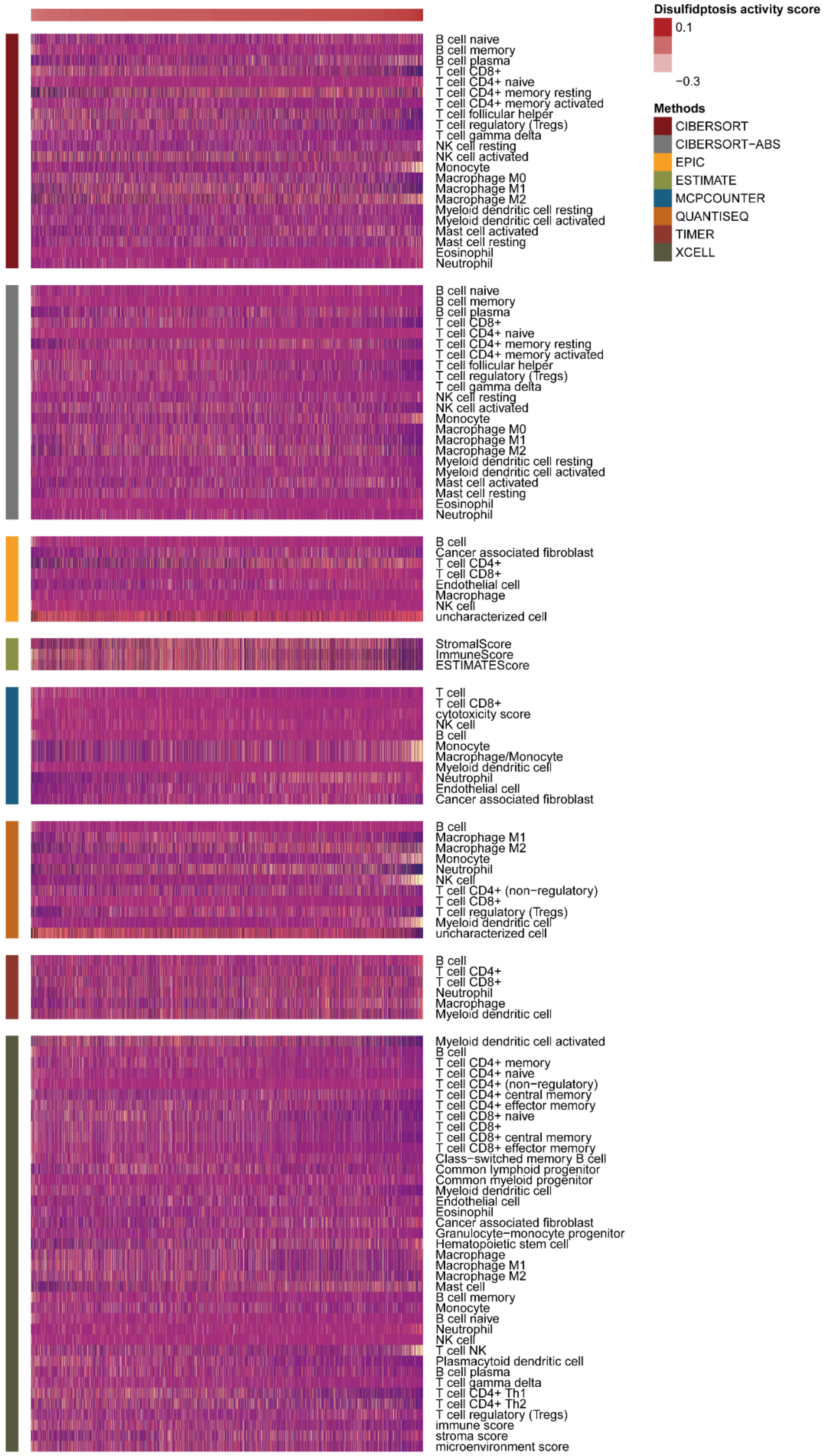

Supplementary Figure S7. heatmap demonstrating the infiltration levels of multiple cells using different algorithms.

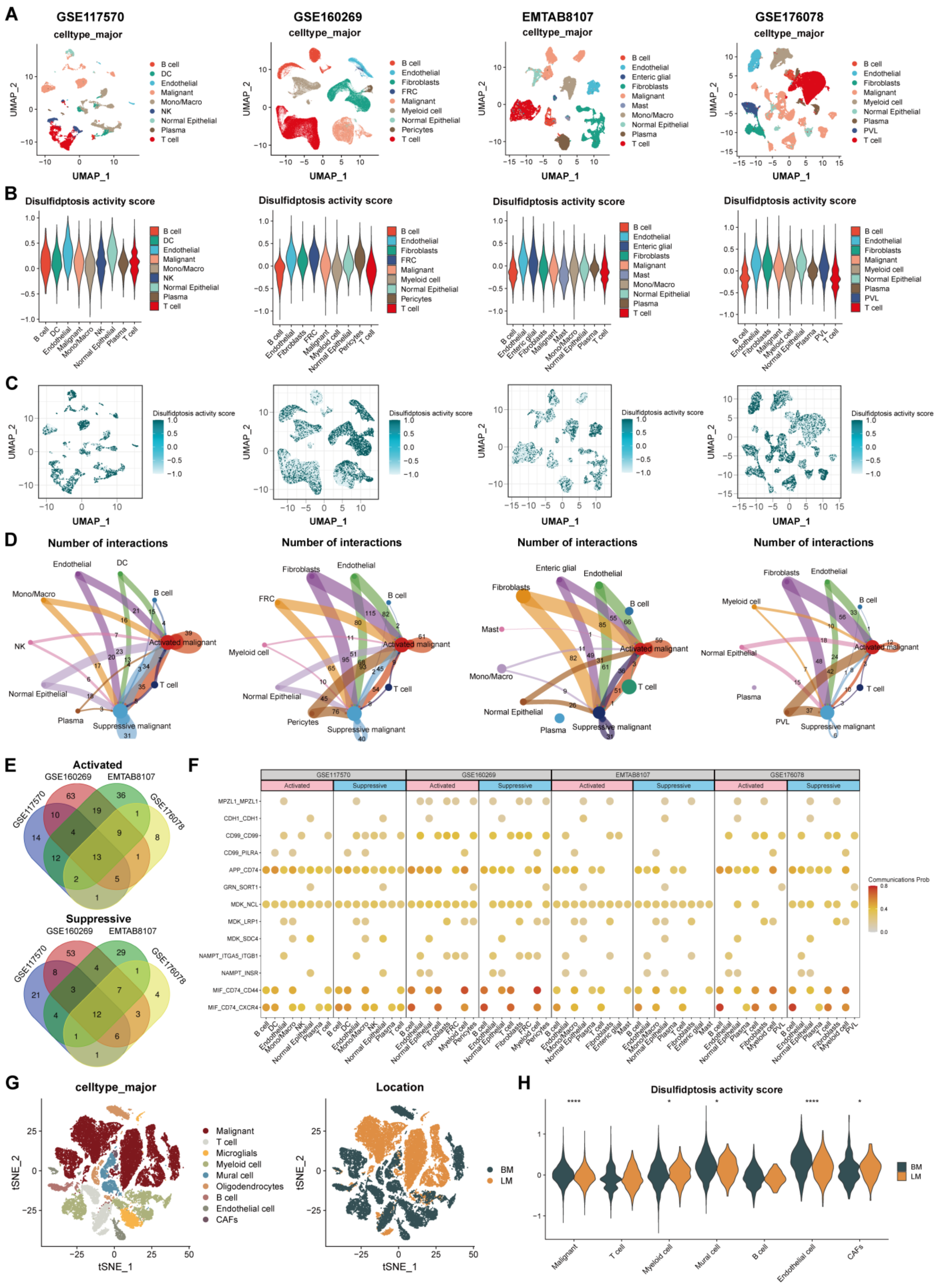
A
GSE117570
celltype_major
GSE160269
celltype_major
EMTAB8107
celltype_major
GSE176078
celltype_major
UMAP_1
UMAP_2
B cell
DC
Endothelial
Malignant
Mono/Macro
NK
Normal Epithelial
Plasma
T cell
Fibroblasts
FRC
Myeloid cell
Pericytes
Enteric glial
Mast
PVL
B
Disulfidptosis activity score
C
Disulfidptosis activity score
D
Number of interactions
Activated malignant
Suppressive malignant
E
Activated
Suppressive
GSE117570
GSE160269
EMTAB8107
GSE176078
F
Activated
Suppressive
MPZL1_MPZL1
CDH1_CDH1
CD99_CD99
CD99_PILRA
APP_CD74
GRN_SORT1
MDK_NCL
MDK_LRP1
MDK_SDC4
NAMPT_ITGA5_ITGB1
NAMPT_INSR
MIF_CD74_CD44
MIF_CD74_CXCR4
Communications Prob
G
celltype_major
Location
tSNE_1
tSNE_2
Malignant
T cell
Microglials
Myeloid cell
Mural cell
Oligodendrocytes
B cell
Endothelial cell
CAFs
BM
LM
H
Disulfidptosis activity score

Supplementary Figure S8. Dissection of disulfidptosis activity score in multiple scRNA-seq cohorts. **(A)** UMAP plot visualization of each scRNA-seq cohort. Different colors refer to different cell types. **(B)** Violin plots of the distribution of the disulfidptosis activity score among different cell types in each scRNA-seq cohort. Different colors refer to different cell types. **(C)** Feature plots of the expression levels of the disulfidptosis activity score in each scRNA-seq cohort. Green indicates high expression, and white indicates low expression. **(D)** Cell-cell interactions between the malignant cells (activated and suppressive clusters) and other different cell types in each scRNA-seq cohort. **(E)** Venn plots of the common ligand-receptor pairs in activated and suppressive groups among scRNA cohorts. (F) Bubble plot of the common ligand-receptor pairs among scRNA cohorts. **(G)** t-SNE plot of single cells profiled in our previous study colored by major cell type and location (BM, brain metastasis; LM, liver metastasis). **(H)** Violin plots of the distribution of the disulfidptosis activity score between different locations in our previous study. * $p < 0.05$, **** $p < 0.0001$.

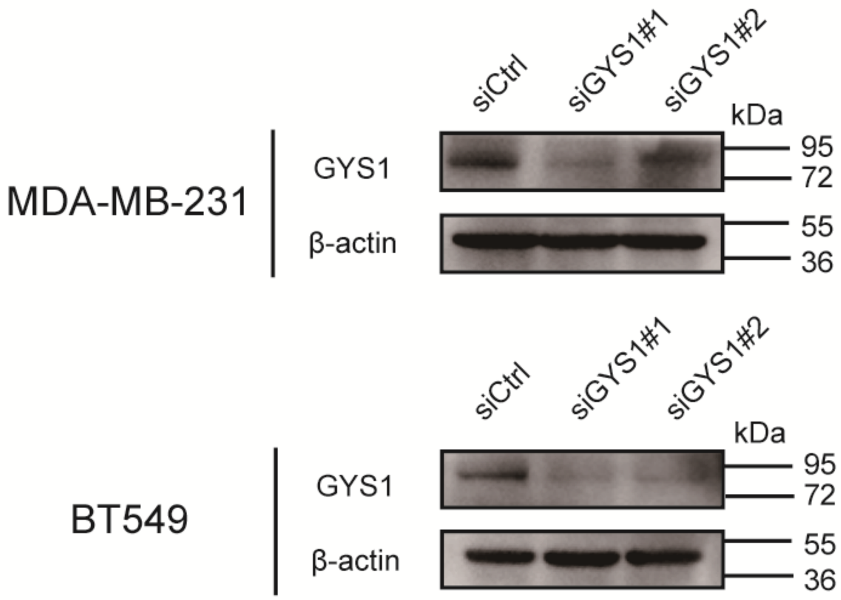

Supplementary Figure S9. Western blot assays showing the efficacy of siRNAs targeting GYS1 in TNBC cell lines.

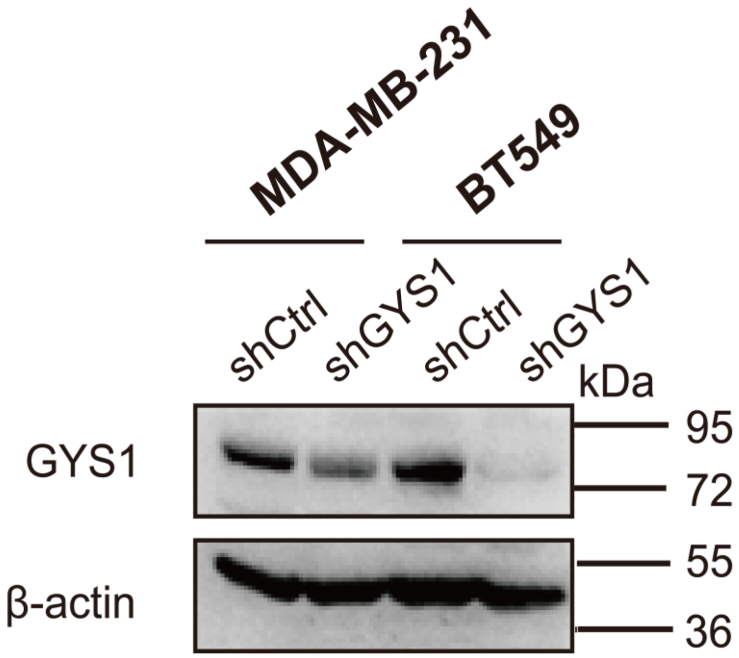

Supplementary Figure S10. Western blot assays showing the efficacy of shRNA targeting GYS1 in TNBC cell lines.

Table S1. Summary of TCGA and GTEx sample sizes of different tumor types in this study.

Abbreviations: ACC, Adrenocortical Cancer; BLCA, Bladder Cancer; BRCA, Breast Cancer; CESC, Cervical Cancer; CHOL, Bile Duct Cancer; COAD, Colon Adenocarcinoma; DLBC, Large B-cell Lymphoma; ESCA, Esophageal Cancer; GBM, Glioblastoma; HNSC, Head and Neck Squamous Cell Carcinoma; KICH, Kidney Chromophobe; KIRC, Kidney Renal Clear Cell Carcinoma; KIRP, Kidney Renal Papillary Cell Carcinoma; LAML, Acute Myeloid Leukemia; LGG, Lower Grade Glioma; LIHC, Liver Hepatocellular Carcinoma; LUAD, Lung Adenocarcinoma; LUSC, Lung Squamous Cell Carcinoma; MESO, Mesothelioma; OV, Ovarian Cancer; PAAD, Pancreatic Cancer; PCPG, Pheochromocytoma & Paraganglioma; PRAD, Prostate Adenocarcinoma; READ, Rectum Adenocarcinoma; SARC, Sarcoma; SKCM, Skin Cutaneous Melanoma; STAD, Stomach Adenocarcinoma; TGCT, Testicular Cancer; THCA, Thyroid Cancer; THYM, Thymoma; UCEC, Uterine Corpus Endometrial Carcinoma; UCS, Uterine Carcinosarcoma; UVM, Ocular melanomas.

Tumor type	TCGA (Tumor)	TCGA (Normal)	GTEx (Normal)
ACC	79	0	258
BLCA	406	19	21
BRCA	1101	113	459
CESC	306	3	19
CHOL	35	9	0
COAD	455	41	779
DLBC	48	0	929
ESCA	163	11	1445
GBM	153	5	2642
HNSC	504	44	0
KICH	65	25	89
KIRC	532	72	89
KIRP	290	32	89
LAML	150	0	0

LGG	513	0	2642
LIHC	371	50	226
LUAD	516	59	578
LUSC	501	49	578
MESO	87	0	0
OV	376	0	180
PAAD	179	4	328
PCPG	181	3	0
PRAD	498	52	245
READ	165	10	779
SARC	260	2	0
SKCM	471	1	1809
STAD	375	32	359
TGCT	134	0	361
THCA	512	59	653
THYM	120	2	0
UCEC	545	35	142
UCS	57	0	142
UVM	80	0	0

Table S2. Datasets enrolled in this study.

Dataset	Tumor types	Sample sizes
TCGA+GTEx	Pan-cancer	23261
GSE62663	NSCLC	8
GSE121378	BRCA	8
GSE183127	LIHC	6
GSE194369	PAAD	10
GSE104462	LIHC	6
GSE32369	COAD	11
GSE206261	PAAD	6
GSE171167	ESCC	8
GSE144833	PAAD	12
CheckMat	ccRCC	181
IMvigor210	BLCA	258
GSE91061	SKCM	28
GSE78220	SKCM	28
GSE58812	BRCA	107
GSE96058	BRCA	143
METABRIC	BRCA	298
GSE21653	BRCA	85
GSE76250	BRCA	66
GSE117570	NSCLC	4
GSE160269	ESCC	60
EMTAB8107	CRC	7
GSE176078	BRCA	9
SYSUCC-Cohort	BRCA	6
FUSCC-Cohort	BRCA	66
Zenodo-ST	BRCA	1

Table S3. siRNAs sequence.

Non-targeting	siControl	5'-UUCUCCGAACGUGUCACGUTT
GYS1	siGYS1#1	5'-CCAACGACGCUGUCCUCUUTT
	siGYS1#2	5'-CCAUCGAGGCACAGCACUUTT

Table S4. shRNAs sequence.

Non-targeting	shControl	TTCTCCGAACGTGTCACGT
GYS1	shGYS1	CCATCGAGGCACAGCACTT

Table S5. Primers for qRT-PCR detection.

GYS1	Forward	GCGCTCACGTCTTCACTACTG
	Reverse	TCCAGATGCCCATAAAAATGGC
β-actin	Forward	CATGTACGTTGCTATCCAGGC
	Reverse	CTCCTTAATGTCACGCACGAT
